# Lipophilic bisphosphonates reduced cyst burden and ameliorated hyperactivity of mice chronically infected with *Toxoplasma gondii*

**DOI:** 10.1128/mbio.01756-24

**Published:** 2024-10-10

**Authors:** Melissa A. Sleda, Zaid F. Pitafi, WenZhan Song, Eric Oldfield, Silvia N. J. Moreno

**Affiliations:** 1 Center for Tropical and Emerging Global Diseases, University of Georgia, Athens, Georgia, USA; 2 Department of Cellular Biology, University of Georgia, Athens, Georgia, USA; 3 School of Electrical and Computer Engineering, University of Georgia, Athens, Georgia, USA; 4 Department of Chemistry, University of Illinois at Urbana Champaign, Urbana, Illinois, USA; Albert Einstein College of Medicine, Bronx, New York, USA

**Keywords:** *Toxoplasma gondii*, bisphosphonate, bradyzoite, hyperactivity

## Abstract

The current treatments for toxoplasmosis are only active against fast-growing tachyzoites, present in acute infections, with little effect on slow-growing bradyzoites within tissue cysts, present in latent chronic infections. The mitochondrion of *Toxoplasma gondii* is essential for its survival, and one of the major anti-parasitic drugs, atovaquone, inhibits the mitochondrial electron transport chain at the coenzyme Q:cytochrome c oxidoreductase site. Coenzyme Q (also known as ubiquinone [UQ]) consists of a quinone head and a lipophilic, isoprenoid tail that anchors UQ to membranes. The synthesis of the isoprenoid unit is essential for cell growth and is inhibited by lipophilic bisphosphonates, which inhibit the parasite growth. In this work, we investigated the effect of lipophilic bisphosphonates on the chronic stages of *T. gondii*. We discovered that three lipophilic bisphosphonates (BPH-1218, BPH-1236, and BPH-1238), effective for the acute infection, were also effective in controlling the development of chronic stages. We showed effectiveness by testing them against *in vitro* cysts and *in vivo* derived tissue cysts and, most importantly, these compounds reduced the cyst burden in the brains of chronically infected mice. We monitored the activity of infected mice non-invasively and continuously with a novel device termed the CageDot. A decrease in activity accompanied the acute phase, but mice recovered to normal activity and showed signs of hyperactivity when the chronic infection was established. Moreover, treatment with atovaquone or BPH-1218 ameliorated the hyperactivity observed during the chronic infection.

**IMPORTANCE:**

Treatment for toxoplasmosis is challenged by a lack of effective drugs to eradicate the chronic stages. Most of the drugs currently used are poorly distributed to the central nervous system, and they trigger allergic reactions in a large number of patients. There is a compelling need for safe and effective treatments for toxoplasmosis. Bisphosphonates (BPs) are analogs of inorganic pyrophosphate and are used for the treatment of bone disorders. BPs target the isoprenoid pathway and are effective against several experimental parasitic infections. Some lipophilic BPs can specifically inhibit the mitochondrial activity of *Toxoplasma gondii* by interfering with the mechanism by which ubiquinone is inserted into the inner mitochondrial membrane. In this work, we present the effect of three lipophilic BPs against *T. gondii* chronic stages. We also present a new strategy for the monitoring of animal activity during disease and treatment that is non-invasive and continuous.

## INTRODUCTION

*Toxoplasma gondii* is a parasitic protist of the phylum Apicomplexa and is the etiological agent of toxoplasmosis. It is estimated that one-third of the world’s population is infected with *T. gondii* (1). In the USA, toxoplasmosis is considered a leading cause of death attributed to foodborne illness and one of five neglected parasitic infections (2). The parasite disseminates throughout the body of the host as fast-replicating tachyzoites, spreading to all organs, including the brain, and eventually converting into bradyzoites within tissue cysts that can remain latent for the duration of the host’s life (3). Tissue cysts containing bradyzoites can be found in all tissues, but they most frequently accumulate in large numbers in neurons of the central nervous system (4). If a host becomes immunocompromised, bradyzoites can revert to tachyzoites and cause severe toxoplasmic encephalitis. The latent chronic infection of toxoplasmosis is correlated with neurological dysfunctions in humans, including schizophrenia, bipolar disorder, epilepsy, rage disorder, and sudden onset psychosis (5). In rodents, it has been shown that the chronic infection leads to varying degrees of central nervous system (CNS) dysfunction that translate into behavioral changes such as hyperactivity, cognitive defects, altered anxiety, and fear responses (6–8). Deciphering the behavioral changes in rodents can lead to insights into the CNS changes that are correlated with infection in humans and can lead to treatments that not only eliminate the cyst burden, but also have an impact on the behavioral changes seen with infection.

Currently, there is no approved treatment for toxoplasmosis that is effective against both the acute (tachyzoite) and chronic (bradyzoite) stages. The most used treatments for toxoplasmosis are the combination of pyrimethamine and sulfadiazine (plus folinic acid), or trimethoprim and sulfamethoxazole. These combinations are highly effective in inhibiting tachyzoite replication, but have no activity on bradyzoites within tissue cysts and therefore do not eliminate the chronic infection (9). Recently, the mitochondrion of *T. gondii* has been shown to be an important target for latent toxoplasmosis. Doggett et al. showed that endochin-like quinolones targeting the mitochondrial cytochrome bc_1_ complex are effective in reducing the cyst burden of chronically infected mice (10, 11). Martynowicz et al. also tested the efficacy of combination therapies against chronic toxoplasmosis using pyrimethamine and an endochin-like quinolone (ELQ-334) to target the mitochondrion (12).

Bisphosphonates (BP) are analogs of inorganic pyrophosphate that are used for the treatment of bone disorders (13). BPs target the synthesis of isoprenoids at several enzymatic steps (14) and they inhibit the growth of several parasite species, including *T. gondii* (15–18). The central enzyme of the isoprenoid synthesis pathway, farnesyl diphosphate synthase (TgFPPS), is not essential for *T. gondii* growth *in vitro* because the parasite can obtain its products, farnesyl diphosphate (FPP) and geranylgeranyl diphosphate (GGPP) (19), from host cells. However, the downstream enzyme that elongates GGPP and FPP into longer isoprenoid units is essential for *T. gondii* growth and can be targeted by lipophilic BPs (20). This long-chain prenyl synthase is also the first enzyme of the synthesis of ubiquinone (UQ) and, because of that, it was named TgCoq1. This enzyme synthesizes the isoprenoid side chain of the UQ molecule, which is essential for its incorporation into the inner mitochondrial membrane (20). Inhibition of TgCoq1 by lipophilic BPs resulted in inhibition of *T. gondii* growth *in vitro* and protection of infected mice with a lethal dose of the parasite.

Lipophilic BPs are analogs of zoledronic acid with hydrophobic side chains added to the imidazole ring (21). Chemically, zoledronate and other BPs are extremely polar and are rapidly removed from blood circulation via binding to bone (22). Unlike zoledronate, lipophilic BPs do not bind to bone material, remain in circulation much longer than conventional BPs, and maintain their inhibitory activity against enzymes of the isoprenoid pathway (21, 23).

In this work, we examined the effect of three lipophilic BPs, previously shown to target the mitochondrion of *T. gondii,* in chronically infected mice, in addition to monitoring their activity throughout the entire 2-month infection period, and compared the effects with a parallel treatment with atovaquone (ATQ). We also tested the effects of these compounds against *in vitro* and *in vivo* produced tissue cysts, and monitored mice non-invasively and continuously during the infection and treatment.

## RESULTS

### Lipophilic bisphosphonates inhibit *in vitro* growth of various strains of *Toxoplasma gondii*

We first evaluated the *in vitro* effect of three lipophilic BPs (BPH-1218, BPH-1236, and BPH-1238) (structures shown in Table 1) that were previously shown to inhibit the first enzyme of the ubiquinone synthesis pathway and impacted mitochondrial function of *T. gondii* tachyzoites (20). We tested these compounds against various strains of *T. gondii* and measured their half maximal effective concentrations (EC_50_s) for inhibition of type I and type II strains. We used red fluorescent parasites of the type II strains, Me49 and Pru, and the type I RH strain. Growth inhibition was measured as described previously, and the EC_50_ values for the compounds were comparable between the three strains (20). For example, BPH-1218 inhibited growth of RH tachyzoites with a 0.3 µM EC_50_, ME49 tachyzoites with a 0.5 µM EC_50_, and Pru tachyzoites with a 0.6 µM EC_50_ (Table 1; Fig. S1). The EC_50_ values for Pru tachyzoites (0.6 µM for BPH-1218 and 10 nM for ATQ) (Table 1; Fig. S1) were comparable to the previously published EC_50_s of these drugs against the type I strain RH (20).

**TABLE 1:** EC_50_s of drugs against various strains of *T. gondii*

Drug	Structure	EC_50_ RH	EC_50_ Pru	EC_50_ Me49	EC_50_ ATQ^R^ R4	EC_50_ ATQ^R^ R5
Atovaquone	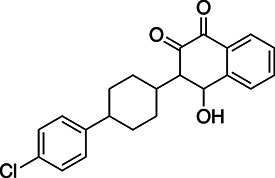	14.1 ± 0.85 nM	4.17 ± 2.34 nM	1.53 ± 0.229 nM	1.87 ± 0.389 µM	1.03 ± 0.29 µM
Pyrimethamine	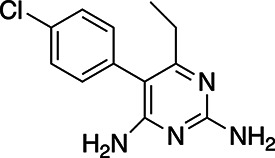	0.383 ± 0.078 µM	43.2 ± 2.58 nM	1.67 ± 0.268 nM	22.1 ± 3.52 nM	5.23 ± 0.221 nM
BPH-1218	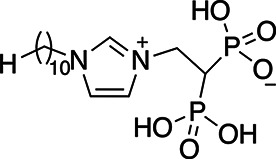	0.372 ± 0.089 µM	0.607 ± 0.187 µM	0.548 ± 0.179 µM	3.12 ± 1.32 µM	2.99 ± 1.19 µM
BPH-1236	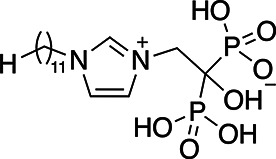	0.77 ± 0.112 µM	0.63 ± 0.113 µM	1.05 ± 0.181 µM	4.53 ± 1.98 µM	4.85 ± 2.31 µM
BPH-1238	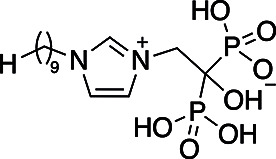	0.701 ± 0.117 µM	0.391 ± 0.166 µM	0.950 ± 0.11 µM	0.66 ± 0.229 µM	1.66 ± 0.243 µM

One of the most important anti-toxoplasma drugs, ATQ, inhibits the mitochondrial electron transport chain at the cytochrome bc_1_ site (24). ATQ is effective mainly against fast-replicating tachyzoites, but it was also shown to be effective for the chronic stages of *T. gondii* in mice (25). Although drug resistance is not a concern for *T. gondii*, ATQ resistant strains (ATQ^R^) are available (24), and we wanted to test the sensitivity of these strains to lipophilic BPs. We used two cell lines previously generated as resistant to ATQ (R4 and R5, from McFadden et al.), (24) and measured the EC_50_s of the three BP compounds and compared them with values found with the parental cell line, Me49. We first tested the sensitivity of the ATQ^R^ strains to ATQ and found that both R4 and R5 showed increased EC_50_s, from 14.1 nM against the RH parasites to 1.9 µM and 1.03 µM, respectively (Table 1; Fig. S1B). However, the BPs showed similar EC_50_s in all cell lines tested (RH, Me49, and Pru), which were not significantly different for the ATQ^R^ cell lines (Table 1).

We previously showed that BPH-1218 protected mice against a lethal acute infection with RH parasites (20). We next evaluated BPH-1236, also a zoledronic acid derivative with a longer alkyl chain (Table 1), for the acute infection with a lethal dose of RH parasites (Fig. S2A). We found that BPH-1236 protected mice against the acute infection (Fig. S2B). We followed disease progression by measuring the mice weight as a function of time and, as expected, mice lost weight at the onset of the acute infection (~9–10 dpi), followed by recovery of the treated ones (Fig. S2C). Control untreated mice became severely ill and either died or were euthanized. Note that one control untreated mouse survived the infection and challenge: it lost weight initially during the acute infection, but recovered. This could be due to this mouse being particularly resistant to the lethal infection, and the evidence indicated that it was infected because of the weight loss and was able to make antibodies to protect itself during the challenge (Fig. S2C and D). The 2 mg/kg treated group had a mouse that started at a lower weight than the majority of the mice, so the error bars for the 2 mg/kg group are larger and the overall weight change is brought down in this group. We challenged the surviving mice with 10,000 tachyzoites at day 30 post-infection (p.i.) and monitored them until they were euthanized, at day 56. All surviving mice had serum antibodies towards the RH-red fluorescent protein (RFP) protein lysate, indicating that they were infected (Fig. S2D).

### Lipophilic bisphosphonates decrease the vacuole size and viability of *in vitro* bradyzoites

**Fig 1:**
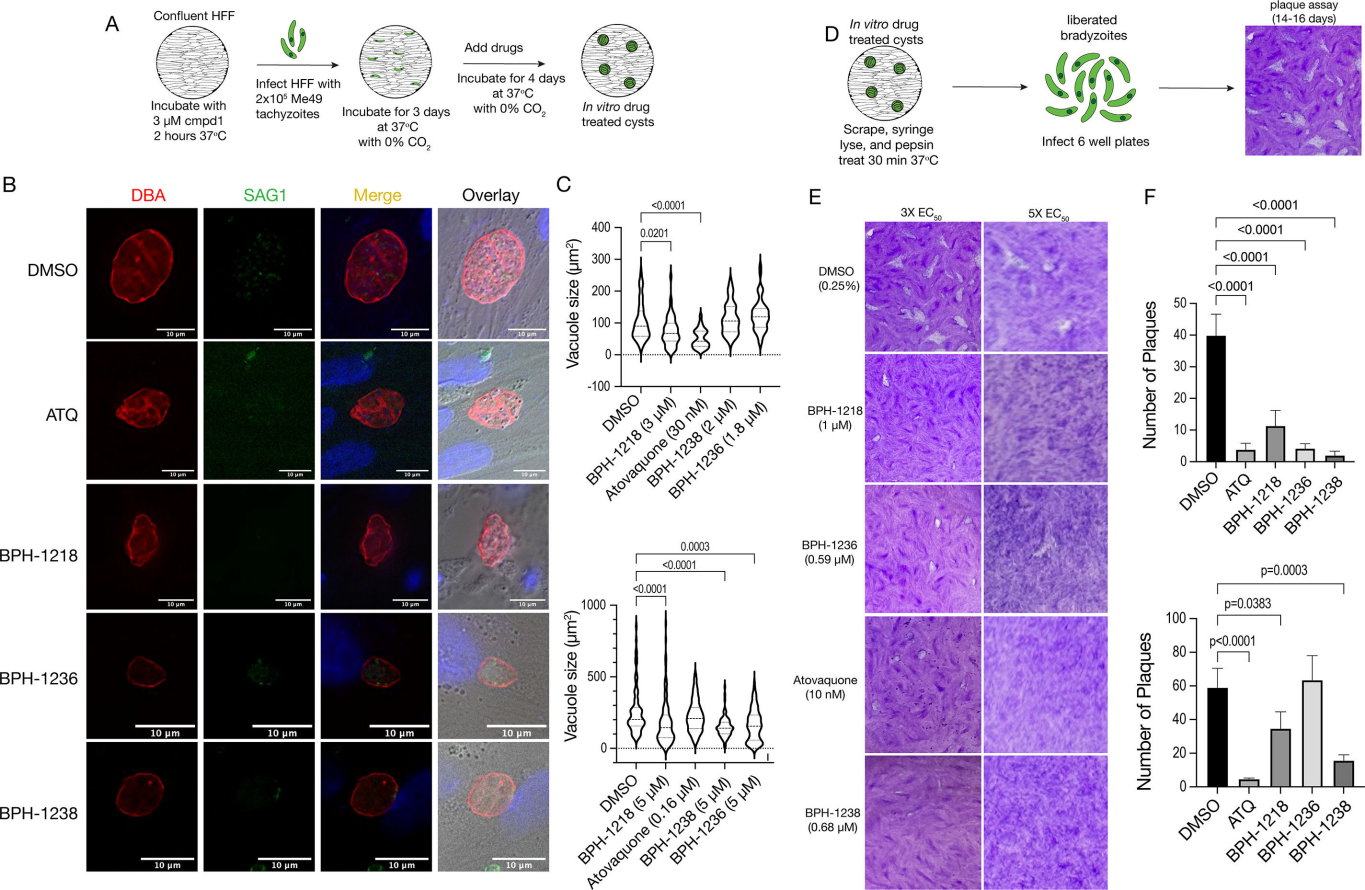
Lipophilic bisphosphonates inhibited the viability of *in vitro* differentiated bradyzoites. (A) Schematic representation of the *in vitro* differentiation of type II Me49 bradyzoites. Human foreskin fibroblast (HFF) cells were incubated with 3 µM compound 1 for 2 hours before infection with 2 × 10^5^ tachyzoites of Me49 followed by incubation at ambient CO_2_ for 3 days. After the tachyzoites differentiated into bradyzoites, compounds were added at the indicated concentrations and incubated for an additional 4 days at ambient CO_2_. At the end of the incubation, cells were fixed, permeabilized, and the cysts stained with DBA and anti-SAG1. (B) Representative images from control and drug-treated cysts with drugs at 5× EC_50_. (C) Quantification of vacuole sizes from cultures exposed to 3 x EC_50_ (top) or 5 x EC_50_ (bottom) concentrations. (D) Schematic representation of the *in vitro* bradyzoite viability assay. Me49 tachyzoites were differentiated into bradyzoites and drug treated as shown in panel A. After drug treatment, the cysts were collected as described in Materials and Methods. HFF monolayers were infected with 10,000 bradyzoites and incubated at 5% CO_2_ for 14–16 days. The plaque image shown is duplicated from Fig. 1E (dimethyl sulfoxide [DMSO] control) and was used as a representative image of plaques. (E) Representative images of plaques formed in the presence of 3× (left panel) and 5× (right panel) the indicated EC_50_s (shown in parentheses). (F) Quantification of the number of plaques from the 3× (top graph) and 5× (bottom graph) EC_50_ incubations. Data for panels C and F are from three independent biological replicates. Two-way analysis of variance statistical analysis was performed to compare the multiple drug-treated groups to the control (DMSO).

To investigate the effect of the three lipophilic BPs against bradyzoites, we first differentiated the type II strains, Me49 or Pru, for 3 days at ambient CO_2_ with compound 1. Compound 1 is a pyrrole, 4-[2-(4-fluorophenyl)-5-(1-methylpiperidine-4-yl)-1H-pyrrol-3-yl]pyridine, which has been shown to dramatically slow the replication of *T. gondii* and is a potent inducer of bradyzoite-specific antigen 1 (26). Three days after differentiation, the *in vitro* cysts were exposed to the BPs for 4 days at ambient CO_2_ (Fig. 1A). After drug treatment, cysts were stained with *Dolichos bifluorus* agglutinin (DBA), which labels the cyst wall, then we determined the vacuole size. All three BPs, BPH-1218, BPH-1236, and BPH-1238, diminished vacuole size when tested at a concentration five times their EC_50_ against tachyzoites (Table 1) and, in addition, BPH-1218 showed the same effect on vacuole size when tested at three times its *in vitro* EC_50_ (against tachyzoites) (Fig. 1B and C). ATQ was used as a positive control and also reduced vacuole size. Representative images of the cysts are shown in Fig. 1B (5× EC_50_) and Fig. S1F (3× EC_50_). In addition, we tested BPH-1218 and ATQ against *in vitro* derived Pru cysts (Fig. S3A) and found that they decreased the vacuole size (Fig. S3B). Pru tissue cysts showed higher expression of the tachyzoite marker SAG1 (surface antifen 1) which is due to the less efficient differentiation into bradyzoites *in vitro*. The SAG1 present inside of the DBA+ cyst is an indication of immature tissue cyst (27). Christiansen et al. showed that under basic pH conditions, type II and III parasites were only able to differentiate up to 73% and 83%, respectively, into mature cysts (27). However, to mitigate this issue, we performed additional experiments investigating the effectiveness of these compounds using *ex vivo* assays with *in vivo* derived tissue cysts treated with acid to remove any potential contaminating tachyzoite (see below).

We next wanted to investigate the viability of bradyzoites exposed to lipophilic BPs. For this, we differentiated and treated cysts as in the previous experiment, but after treatment, we collected bradyzoites, released them from cysts by syringe passage, and treated them with pepsin, to remove any remaining tachyzoites. Then, we plated the bradyzoites on a prepared culture of fibroblasts to allow the parasites to form plaques in normal growth conditions, undisturbed for 14 or 16 days (Fig. 1D). At the end of this experiment, we enumerated the number of plaques and compared the results with the DMSO control. We found that BPH-1218, BPH-1236, and BPH-1238 treated bradyzoites were less viable than the dimethyl sulfoxide (DMSO) control, since the plaque number was significantly reduced (Fig. 1E and F). Representative images of the plaques are shown in Fig. 1E.

All the previous work was completed using a type II cystogenic strain, so we next tested the effects of the lipophilic BPs on a type I/III cystogenic strain (EGS, strain isolated from human amniotic fluid) that spontaneously differentiates in culture to form cysts (28, 29). This allowed us to test the effects of the drugs on cysts that are spontaneously formed, avoiding the use of pH or chemical stress with compound 1. The EGS strain takes approximately 96 hours to spontaneously form cysts, and we exposed them to the drugs at this time for an additional 4 days before fixing the cultures and performing an immunofluorescence assay using DBA and anti-SAG1 antibodies (tachyzoite control) (Fig. S4A). Example images of the cysts treated with BPH-1218, BPH-1236, BPH-1238, and ATQ compared to the DMSO treated controls are shown in Fig. S4B. We also quantified the size of the cysts and found that drug treatments (ATQ, BPH-1218, BPH-1236, and BPH-1238) reduced the size of the vacuoles at concentrations three times their EC_50_ (Fig. S4C).

Taken together, our results showed that lipophilic BPs decreased the vacuole size and viability of *in vitro* derived cysts.

### Lipophilic bisphosphonates inhibit the growth and viability of *in vivo* derived tissue cysts

**Fig 2:**
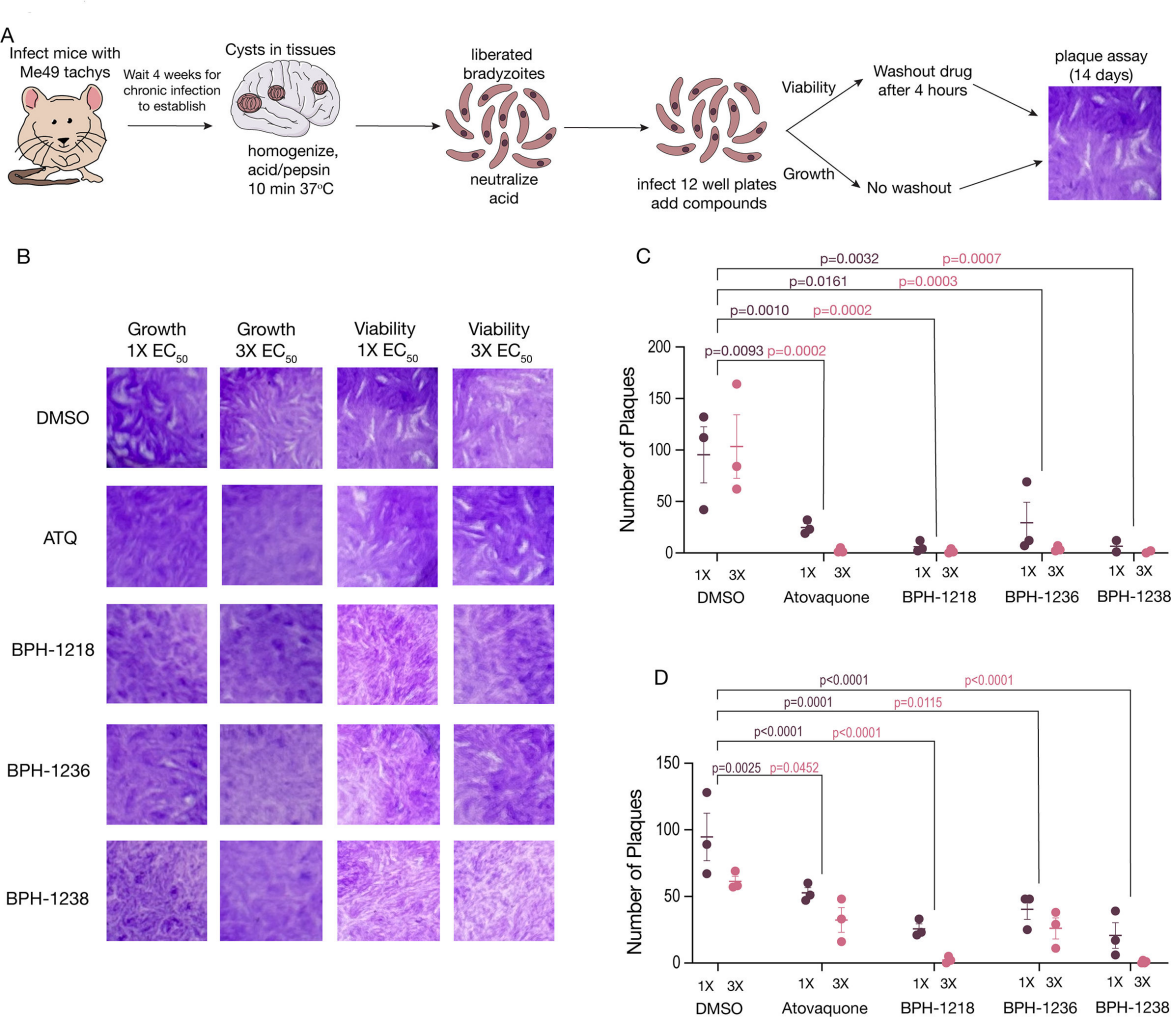
Lipophilic bisphosphonates inhibited growth of *ex vivo* derived bradyzoites and reduced tissue cyst size. (A) Schematic representation of the *ex vivo* bradyzoite collection. Brains of chronically infected mice were collected, homogenized, and treated with acid/pepsin to release bradyzoites. The liberated bradyzoites were plated on human foreskin fibroblast (HFF) monolayers and treated with drugs for 14 days (growth) or for 4 hours (viability). The plaque image shown is duplicated from Fig. 2B (DMSO control) and was used as the representative image of plaques. (B) Representative images of plaques from *ex vivo* growth and viability from Me49-RFP. (C) Quantification of the number of plaques from the *ex vivo* growth assay of Me49-RFP at 3× the EC_50_. (D) Quantification of the number of plaques from the *ex vivo* viability assay of Me49-RFP at 3× the EC_50_. Data from panels C and D were from three independent biological replicates. Two-way analysis of variance statistical analysis was performed to compare the multiple drug-treated groups to the control (DMSO). Values in panels C and D are means ± SEM.

Considering that *in vitro* derived bradyzoites differ from the *in vivo* derived ones, we tested the effects of lipophilic BPs on bradyzoites isolated from brain cysts. For this, we collected tissue cysts from infected mice and performed *ex vivo* growth and viability experiments. We collected tissue cysts from the brains of chronically infected mice 28 days post-infection, then homogenized and treated them with pepsin, to release the bradyzoites and remove any remaining tachyzoites. For growth inhibition experiments, we plated the released bradyzoites on confluent fibroblast cultures to allow the formation of plaques for 14 days, in the presence of inhibitors. For viability inhibition experiments, the released bradyzoites were plated in the presence of inhibitors for 4 hours and, at this time, the media was replaced with regular Dulbecco’s modified eagle medium (DMEM) and parasites were allowed to grow for 14 days (Fig. 2A). Representative images of *ex vivo* plaques are shown in Fig. 2B. All the inhibitors decreased the number of plaques (Fig. 2C and D). BPH-1218, BPH-1236, and BPH-1238 reduced the viability of *ex vivo* bradyzoites, as is evident in the reduction of the number of plaques formed with the highest difference at three times the *in vitro* EC_50_ (Fig. 2D). Interestingly, ATQ was effective against bradyzoite growth, but had only a minimal impact on viability. We also performed *ex vivo* growth and viability experiments with bradyzoites isolated from chronically infected mice with the Pru strain (Fig. S3C). We found a significant reduction in the number of plaques formed at three times the EC_50_ for ATQ, BPH-1218, BPH-1236, and BPH-1238 for both growth (Fig. S3D) and viability (Fig. S3E). Taken together, these results showed that BPs reduced the viability of bradyzoites from both type II strains, Me49 and Pru.

### Lipophilic bisphosphonates decrease the cyst size and cyst burden in chronically infected mice

**Fig 3:**
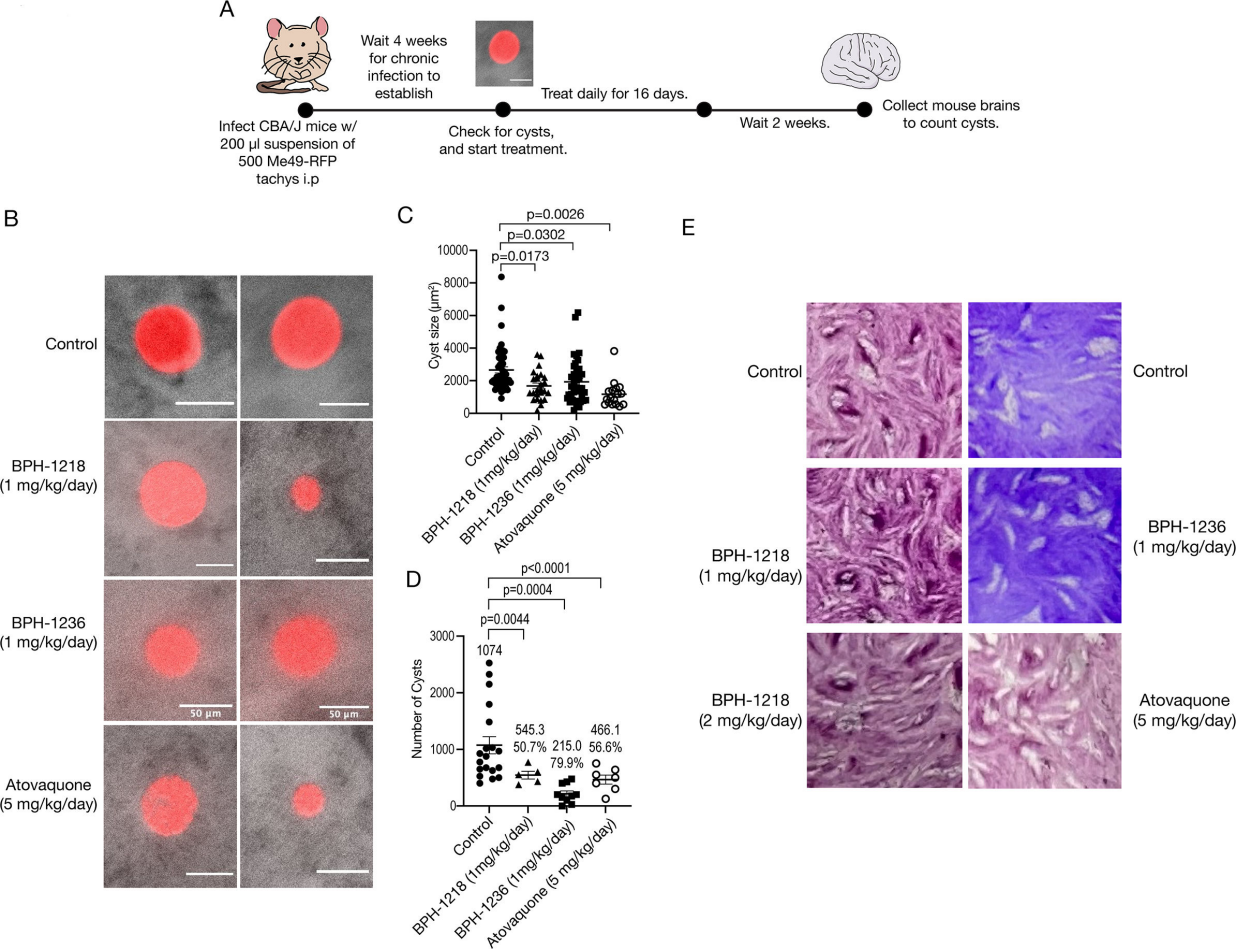
Lipophilic bisphosphonates reduced the cyst burden of chronically infected mice. (**A**) Schematic representation of the infection for treatment of chronically infected mice. CBA/j mice were infected i.p. with 500 Me49-RFP tachyzoites. After 28 days post-infection, two mice were checked for cysts and the remaining mice treated daily for 16 days. After 2 weeks, the brains were collected, and cysts were quantified. (B) Representative images of cysts isolated from control and drug-treated mice. (C) Quantification of cyst size. (D) Quantification of the number of cysts. (E) Representative images of plaques from *ex vivo* bradyzoites from the control and drug-treated mice. Data from panels C and D are from three independent biological replicates. Two-way analysis of variance statistical analysis was performed to compare the multiple drug-treated groups to the control.

To investigate the potential protective effect of lipophilic BPs on the *T. gondii* chronic infection, we infected CBA/j mice intraperitoneally (i.p.) with 500 Me49-RFP tachyzoites and allowed the chronic stages to get established for 4 weeks. At this time, the brains of two mice were analyzed for the presence of tissue cysts, and the remaining mice were treated for 16 days with the indicated compounds at the indicated doses (Fig. 3). The brains of all mice were collected and the tissue cysts enumerated, 2 weeks after the end of the treatment (Fig. 3A). A high variability in cyst size was found for all groups, including for the controls (Fig. 3B); however, the size of the cysts from the treated mice was significantly smaller than those of the control group (Fig. 3C). BPH-1218 showed a 50.7% reduction in the number of cysts compared to the control, while the positive control (ATQ) showed 56.6% reduction (Fig. 3D). The greatest decrease was seen with BPH-1236, which reduced the number of cysts by 79.9%, compared to the control (Fig. 3D). Note that the doses for these compounds was 1 mg/kg. We next questioned if the remaining drug-treated tissue cysts were still viable after treatment, and for this, we plated the *in vivo* derived tissue cysts in an *ex vivo* tissue culture assay and allowed them to form plaques without any *in vitro* treatment, finding that the remaining bradyzoites were still viable (Fig. 3E).

We also tested the type II Me49-RFP strain in Swiss Webster mice. For this, we infected Swiss Webster mice with 5,000 Me49-RFP parasites i.p. and followed the same timeline as the infection of CBA/j mice (Fig. S5A). Interestingly, only ATQ reduced the size of the cysts (Fig. S5C). Most importantly, we found that BPH-1218 and ATQ reduced the cyst burden of the chronically infected Swiss Webster mice by 73.6% and 65.5%, respectively (Fig. S5D). The weight was also monitored during infection, and after an initial decrease during the acute phase of infection, the mice continually gained weight for the duration of the experiment (Fig. S5E). BPH-1218 and BPH-1236 are, therefore, effective against the chronic infection *in vivo* and can reduce the cyst burden in mice.

### Continuous and non-invasive monitoring of mouse activity

**Fig 4:**
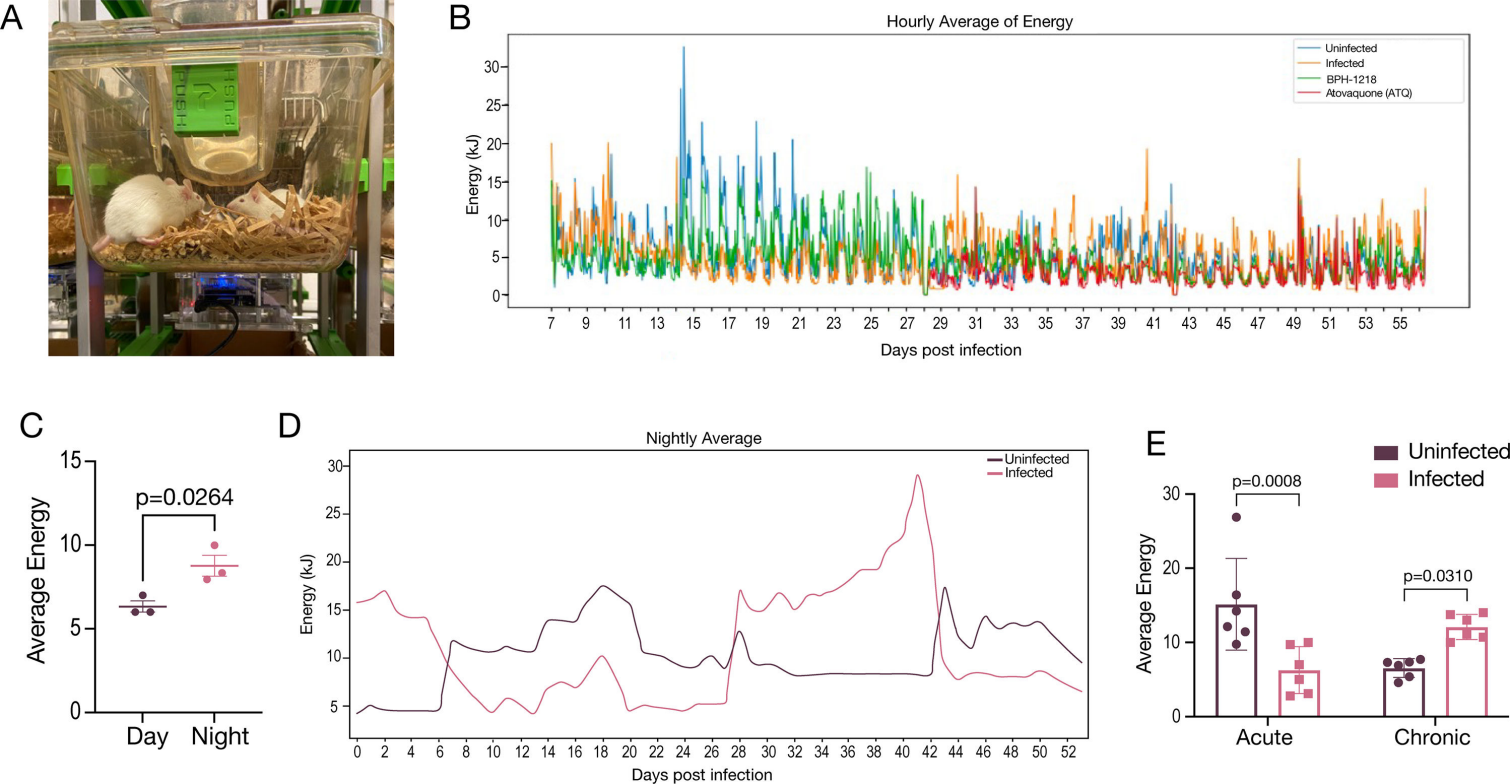
Continuous monitoring of mice activity revealed hyperactivity during chronic infection. (A) Image of the CageDot device attached to the bottom of the mouse cage. (B) Continuous tracing for the entire experiment 1 (56 days) showing the hourly average for each cage. The traces represent the day and night cycles for each cage for all days of the experiment. Day and night data were used for the analysis presented in panel C, and night data were used for all the other analyses. Each cage contained four mice, and the data are the average for all of the mice in the cage. (C) Quantification of energy averages between daytime vs night time (4-hour period for each) for the uninfected cage showing the changes between night and day. (D) Continuous tracings of uninfected and Pru-GFP-infected Balb/c mice for a 54-day experiment (GFP, green fluorescent protein). The acute data represent the activity computed at days 10–20, and the chronic stage was from data collected at days 30–40. Some preliminary monitoring results were previously communicated as an example for the use of the CageDot (31) . (E) Quantification of the acute and chronic stage of disease for uninfected and infected mice. Average of three biological replicates. Values in panels C and E are means ± SEM. Student’s *t*-test statistical analysis for data in panels C and E.

With the aim to monitor activity of mice during the disease and treatment, we used a device named the CageDot (30, 31), which is a sensor that collects vibration data and calculates the energy of the animals which correlates to the activity. We attached the CageDot sensor to the bottom of the cage (Fig. 4A). This ensures that the monitoring is non-invasive and would not alter the activity of the mice. We allowed the CageDot sensor to continuously monitor the activity for the duration of the experiment. We show in Fig. 4B the hourly average of energy for each cage during the duration of the first experiment. The traces represent the day and night cycles for each cage for all days of the experiment. We detected clear differences in the energy between day (6.3) and night (8.34) in the uninfected mice, which was 28% (*P*: 0.03) increased during the night (Fig. 4C). This was expected as mice are nocturnal animals. For all further calculations of activity, we used the data from the nightly averages since this is when the animals were awake. We compared the activity between infected and uninfected mice during acute and chronic stages for 54 days (Fig. 4D). The mice went through an acute phase of the infection at days 10–20, and we confirmed the presence of cysts at 28 dpi and evaluated the chronic activity at days 30–40. We saw a significant reduction in activity in the infected mice during the acute phase (Fig. 4E), which corresponded to the mice getting ill from the infection. The infected mice also lost weight compared to the control (Fig. S6A and B). After ~20 days post-infection, mice showed recovery in their activity, and right before the start of treatment (~26 dpi), we evaluated the activity of the chronically infected mice and observed that the infected mice had a 46% increase in activity compared to the uninfected controls (Fig. 4E). This increase in activity is a characteristic previously observed during chronic infection with *T. gondii* (12). To our knowledge, this is the first report of a continuous and non-invasive monitoring of disease progression in mice infected with *T. gondii*, and using this approach, we confirmed that *T. gondii* infection causes hyperactivity in mice during the chronic phase.

**Fig 5:**
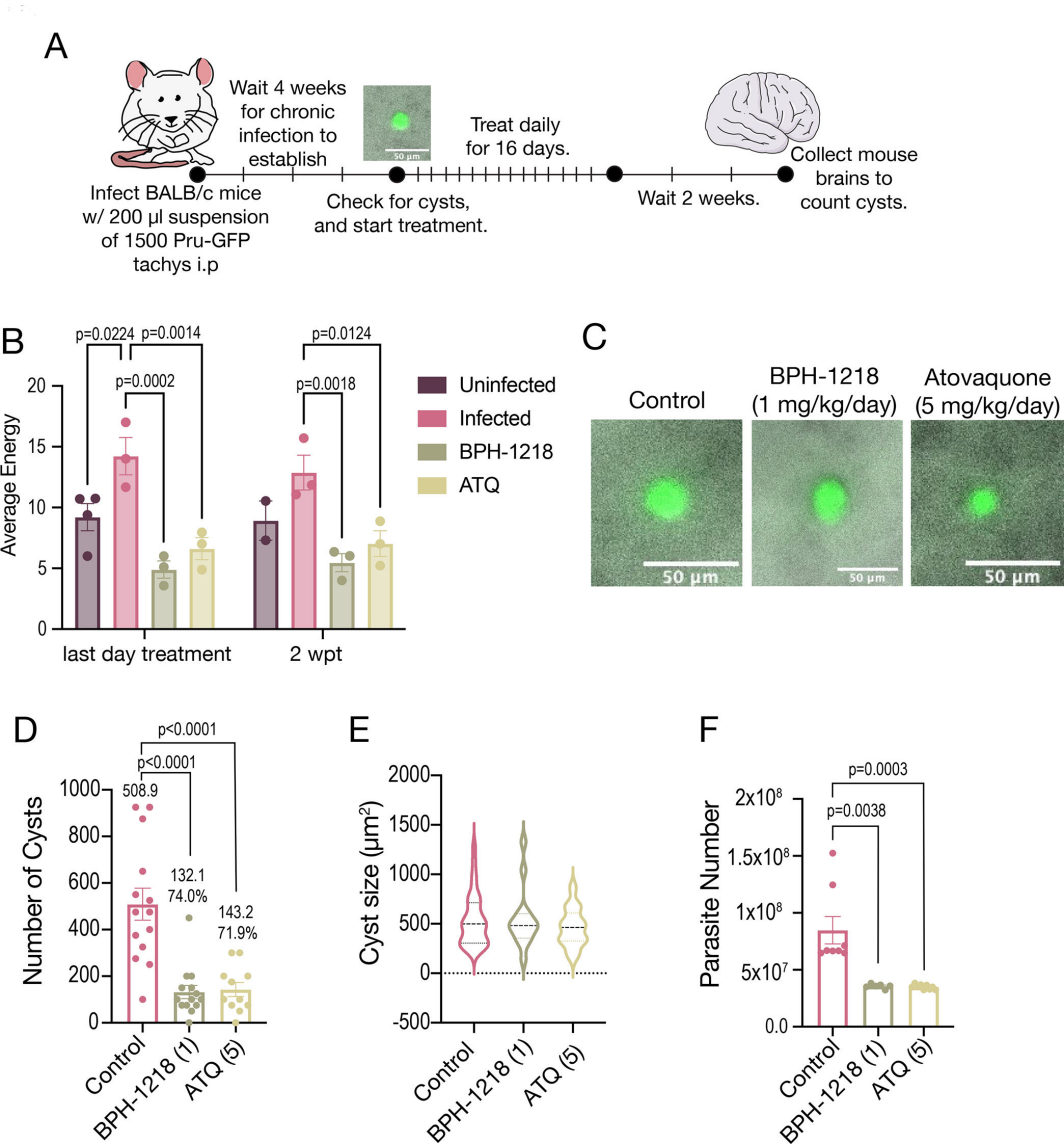
The hyperactivity of chronically infected mice was reduced upon treatment with BPH-1218. (A) Model for chronic infection. Balb/c mice were infected i.p. with 1,500 Pru-GFP tachyzoites. After 28 days, two mice brains were checked for tissue cysts and the rest were treated daily for 16 days. Two weeks after the end of treatment, the brains were collected and cysts enumerated. (B) Nightly average energy from the last day of treatment and 2 weeks post-treatment. (C) Representative images of Pru-GFP cysts from the control and drug-treated mice. (D) Quantification of the cysts from the control and drug-treated mice. (E) Quantification of the cyst size from the control and drug-treated mice. (G) RT-PCR quantification for parasite tubulin and normalized to parasite number. Data from panels B, D, E, and F are averages from three independent biological replicates. Except for panel B, the uninfected cage at 2 wpt had only two replicates due to a recording error with the CageDot device. Two-way analysis of variance statistical analysis was performed where statistics are shown.

Chronic toxoplasmosis has been linked to behavioral changes in mice, as well as neurological disease in humans (32). We thus next sought to utilize the continuous monitoring approach (CageDot) to investigate the hyperactivity of chronically infected mice, and the impact of experimental treatments. For these experiments, we infected Balb/c mice i.p. with 1,500 Pru-GFP (green fluorescent protein) tachyzoites and monitored mice activity for the entire infection period. At 28 dpi, we collected brains from two mice to check for the presence of tissue cysts and started daily treatment for 16 days. Two weeks post-treatment, we collected the brains of all animals and counted cysts (Fig. 5A). We then looked at the nightly averages in activity for the duration of the experiment. The cages with infected mice showed a 58.6% decrease in activity compared to the uninfected during days 14–20 which corresponded with the phase of acute disease. The uninfected mice showed an increase in activity which could be due to external factors of the mice responding more actively when they were handled to weigh them. This was followed by a steady decline as they likely became familiar with being handled more frequently. Approximately 30% of the infected mice died during the acute infection, but we kept the cages with the same number of mice by replacing them with extra infected mice. This was important for the consistency of the motility data. At 28 dpi, we confirmed the presence of cysts in the brains of two mice and started daily i.p. treatment for 16 days with BPH-1218 at 1 mg/kg/day or ATQ at 5 mg/kg/day. At the end of the treatment (15 days post-treatment [dpt]) and 2 weeks post-treatment (2 wpt), we observed that the infected control mice still showed signs of hyperactivity compared to the uninfected controls (Fig. 5B). We also found that treatment with both BPH-1218 or ATQ decreased the activity of the infected mice to similar levels of the control uninfected mice (Fig. 5B). We collected the brains of the mice at the end of the experiment, and representative images of cysts are shown in Fig. 5C. We also determined that BPH-1218 and ATQ reduced the cyst burden in the Balb/c infected with Pru-GFP model of chronic infection, by 74.0% and 71.9%, respectively (Fig. 5D). The cysts in the control versus the drug-treated groups had similar sizes (Fig. 5E). We measured tubulin mRNA expression in the brains of these mice using reverse transcription (RT)-PCR and normalized to the parasite number of the Pru-GFP strain. We found an overall decrease in the number of parasites (tubulin) in the drug-treated mice (Fig. 5F). At the end of the experiment, we also collected serum from the mice to test for the presence of antibodies against Pru-GFP total lysates and found that all mice that were infected showed antibodies against Pru-GFP, indicating that their survival and subsequent reduction in cyst number were from the drug treatment (Fig. S6C). Taken together, our results showed that behavioral changes like hyperactivity caused by the infection with *Toxoplasma gondii* can be monitored non-invasively and continuously, and this hyperactivity is ameliorated by treatment with the lipophilic BP, BPH-1218, or ATQ.

## DISCUSSION

In this work, we tested the effectiveness of the lipophilic BPs, BPH-1218, BPH-1236, and BPH-1238 against the chronic stages of various strains of *T. gondii* (RH, Pru, Me49, EGS, and ATQ^R^) using *in vitro* and *in vivo* models. We tested the effects of the BPs against *in vitro* derived cysts and showed that they were able to decrease the size and viability of both Me49 and Pru strain tissue cysts. We also found that they were effective at reducing the size of *in vitro* derived cysts of the type I/III EGS strain. Reduced tissue cyst size most likely is the result of reduced number of bradyzoites, which means that these drugs are affecting their proliferation. These lipophilic BPs also reduced growth and viability of *in vivo* derived tissue cysts in an *ex vivo* assay. BPH-1218 and BPH-1236 were tested in a chronic infection model in mice (infecting Swiss Webster and CBA/j mice with Me49-RFP) and found that both BPH-1218 and BPH-1236 decreased cyst burden. We monitored activity of infected mice using a novel strategy based on the use of a non-invasive and continuous protocol with a new device called the CageDot (30, 31 ).With this device, it was possible to follow the whole course of the disease, monitoring mice activity throughout both acute and chronic phases of the infection (Balb/c mice infected with Pru-GFP). When infected mice go through the acute infection, there was a decrease in activity, and when they transitioned to the chronic phase (~28 dpi), there was an increase in activity (hyperactivity), compared to uninfected controls. Upon treatment with BPH-1218 or ATQ, we observed amelioration of the hyperactivity during the chronic phase at comparable levels to the uninfected mice. This reduction of the hyperactivity by BPH-1218 and ATQ was accompanied by a significant decrease of the cyst burden.

The current treatments for toxoplasmosis (sulfa drugs and pyrimethamine) are effective against the fast-replicating tachyzoites with very little effect against the bradyzoites found in tissue cysts. Recent research has shown that targeting the mitochondria of bradyzoites (i.e., endochin-like quinolones) reduced the cyst burden in chronically infected mice (10). There is still work that needs to be done to characterize the mitochondrial activity in the bradyzoite stage; however, there are many challenges to studying this in bradyzoites because of the difficulty of isolating large numbers of parasites. A recent study characterized the morphology of the mitochondrion of a small number of tissue cysts using a machine learning algorithm (33). This approach utilized a MitoTracker dye, which indicated that the mitochondria in bradyzoites have a significant membrane potential which can be an indication of the metabolic state of the cells. Our results indicate that tissue cysts may have an active mitochondrion, and that it is possible to target the mitochondrial ubiquinone biosynthesis pathway as a strategy for controlling chronic toxoplasmosis

Our lab has characterized enzymes of the isoprenoid pathway including TgFPPS (34, 35) and TgCoq1 (20) and found that they can be inhibited by BPs. TgCoq1 was responsible for determining the side-chain length of UQ, which is important for anchoring UQ to the inner mitochondrial membrane for its function in the electron transport chain, shuttling electrons from complex 2 to complex 3. In this work, we tested inhibitors (BPH-1218 and BPH-1236) of the TgCoq1 enzyme and showed that targeting this activity is a valid strategy to reduce cyst burden in chronically infected mice. These lipophilic BPs also inhibit TgFPPS (Table 2 in reference 20) and this double-hit may result in a synergistic effect on cell growth inhibition. Moreover, these compounds also inhibit human FPPS as well as human GGPPS (21), and since we previously showed that inhibiting the host isoprenoid pathway was a valid strategy to block *T. gondii* growth (19, 36), a host-directed effect may also contribute to overall anti-parasitic activity. Additionally, lipophilic BPs have improved pharmacokinetic properties compared to zoledronate and other bone-targeting BPs, which are polar and are cleared from circulation because they bind to the bone (37). Lipophilic BPs have almost no affinity for bone (37) and can remain in circulation much longer than, e.g., zoledronate (21). Altogether, these features make the lipophilic BPs promising chemotherapeutics for chronic toxoplasmosis.

ATQ is one of the clinically approved treatments for toxoplasmosis. Previous studies and our data showed that ATQ is able to reduce cyst burden in chronically infected mice (25). Our *ex vivo* assays showed that ATQ was effective against tachyzoite replication, but had a minimal impact on the viability of the bradyzoites. This observation suggests that the reduction in cyst burden could be the result of ATQ’s effect on the tachyzoites that result from bradyzoite reactivation. It has been shown that ATQ is active against tachyzoites and tissue cysts, both *in vitro* and *in vivo* (25, 38–41). However, clinical trials have shown mixed results with ATQ being unable to prevent relapse of infection, with up to 75% of patients experiencing relapse after 6 years of therapy (42, 43). This prolonged treatment and great potential for relapse indicated that ATQ is unable to eliminate all tissue cysts. Therefore, there is a need for a more effective treatments of the chronic stages of toxoplasmosis.

The infection with *T. gondii* results in a chronic latent disease that persists in immunocompetent individuals. Recent work was aimed at understanding the latent and persistent forms of the disease (44). Previous experimental drug testing against chronic stages of *T. gondii* showed up to ~90% clearance of tissue cysts from the brains of infected mice (10, 12, 45). However, very little is known about the characteristics of the cysts that persist after treatment. We also saw a persistent population of cysts with BP treatment, and we found that they are still viable as they are still able to grow in culture.

*Toxoplasma gondii* causes a long-term latent infection in the CNS of humans and rodents. There have been previous studies investigating how the infection with *T. gondii* can lead to changes in rodent and human behavior (46). Our study utilized a sensor that can continuously monitor mouse activity throughout the entire 58-day experiment. We observed differences during the acute, chronic, and treatment (with BPH-1218) phases of the infection. This approach eliminated the issue of the timing of the measurement as it evaluates activity throughout and will eliminate some of the inconsistencies between the different behavioral assays by monitoring non-invasively in the mouse’s natural home-cage environment. In the future, it will be interesting to test other strains of rodents and parasites with this approach.

More work is needed to clarify the level of activity of the bradyzoite mitochondrion, but considering previous results showing evidence for the presence of a membrane potential combined with the effect of mitochondrial inhibitors against bradyzoites in cysts, it is possible to infer that the mitochondria is a good target for treating the chronic infection of *Toxoplasma gondii*. It was less clear how viable the persistent cysts were, and if reactivated, would they lead to the death of infected mice? We demonstrated with the *ex vivo* assays of drug-treated mice that the remaining cysts are still viable, but the reason for their persistence is unknown. In conclusion, we presented a new target, TgCoq1, for the treatment of the chronic infection of toxoplasmosis and several potential effective compounds. We also presented a new strategy for the monitoring of animal activity that is non-invasive and continuous that can be used in various animal-study applications.

## MATERIALS AND METHODS

### Chemicals and reagents

Oligonucleotide primers were obtained from Integrated DNA Technologies. TRIzol Reagent and Superscript III Reverse Transcriptase were from Invitrogen. Deoxynucleotide triphosphates (dNTPs) were from New England BioLab Inc. Other reagents were analytical grade or better.

### Cultures

*T. gondii* RH strain (all type I strains) were cultured using hTERT (human telomerase reverse transcriptase) cells with 1% bovine calf serum (BCS) and purified as described earlier (47). *T. gondii* Me49, Pru, and EGS strains (all type II and I/III strains) were cultured using human foreskin fibroblast (HFF) cells with 1% BCS. EGS strain is from BEI Resources (https://www.beiresources.org/). The Me49 parental strain was transfected with a td-Tomato plasmid to create the Me49-RFP strain. The Pru-GFP and Pru-RFP strains were a gift from Laura Knoll. Host cells were grown in Dulbecco’s modified Eagle medium supplemented with 10% BCS (hTERT) and 10% fetal bovine serum (FBS) (HFF). Cell cultures were maintained at 37°C with 5% CO_2_.

### *In vitro* drug screening and growth assays

Experiments with *T. gondii* tachyzoites were carried out with parasites expressing a td-Tomato RFP (48, 49). For drug testing and growth assays, parasites were purified by passing them through a 25-gauge needle, followed by filtration through a 5 µm filter. Human fibroblasts were cultured in 96-well black plates for 48 (hTERT) or 120 (HFF) hours prior to the addition of 4,000 (type I) or 8,000 (type II) fluorescent tachyzoites per well. Fluorescence values were measured for up to 7 days (type I) and 21 days (type II), and both excitation (544 nm) and emission (590 nm) were read from the bottom of the plates in a Molecular Devices plate reader (36). The EC_50_s were calculated using GraphPad Prism software.

### Testing of lipophilic BPs in mice infected with a lethal dose of *T. gondii*

Experiments were carried out as described previously (16) using 100 *T. gondii* tachyzoites of the RH td-Tomato strain (RH-RFP) to infect female Swiss Webster mice. Drugs were dissolved in 10% Kolliphor HS-15 and were inoculated i.p. starting 6 hours after infection and administered for 10 days. The survival of mice was monitored along with their weight. Mice were sacrificed if they lost over 20% of their body weight. Surviving mice were challenged on day 30 post-infection with 10,000 RH-RFP tachyzoites. Blood was collected at 30 dpi (before challenge) and at the time of euthanasia (3 weeks post challenge) to analyze the presence of *T. gondii* antibodies. To test for antibodies, we ran a western blot with RH-RFP lysate protein and probed the membrane with the serum collected from the mice. Protein extraction and western blot conditions were as done previously (20). After blocking with 5% milk in PBS-T (0.1% Tween 20 in phosphate buffered saline [PBS]), we washed the membrane three times for 5 minutes with PBS-T and attached it to the BioRad multiscreen tool. The RH-RFP lysate was ran throughout the whole gel to allow for testing of different serum (antibodies) on the same membrane. We incubated with the mouse serum (“primary antibody”) for 1 hour in the multiscreen tool and then washed the membrane five times for 3 minutes each while the membrane was still in the multiscreen wells and an additional wash after removing the membrane. Then, we used IRDye 800CW goat anti-mouse IgG secondary antibody and incubated for 1 hour before washing three times with PBS-T and imaging the blot on a Licor Odyssey CLx machine at 800 nm.

### *In vitro* differentiation of bradyzoites and immunofluorescence assays (IFA)

Type II Me49 or Pru was differentiated using compound 1 as previously described (50). Confluent HFF monolayers on glass coverslips (preincubated for 2 hours with 3 µM compound 1, as described previously [26]) were infected with 5 × 10^3^ tachyzoites of the ME49 or Pru (type 2) or EGS (type I/III) strain (no compound 1 for EGS). The cells were allowed to grow and differentiate in a 37°C incubator with ambient CO_2_ for 3 days (EGS was allowed to spontaneously differentiate for 4 days). The cells were then incubated with drugs at three times their EC_50_ for an additional 4 days at ambient CO_2_. After 7 days (Me49) or 8 days (EGS) of incubation, the cells were fixed with 3% paraformaldehyde for 10 minutes, permeabilized with 0.25% Triton X-100 for 10 minutes, and then blocked in 3% bovine serum albumin for 1 hour. The coverslips were then incubated in DBA rhodamine (for Me49)(51) or DBA lectin (a kind gift from Diego Huet for EGS) and anti-SAG1 rat antibody (a kind gift from Vern Carruthers) for 2 hours. Then, the secondary anti-rat 488 was used to visualize the SAG1 antibody. The cells were then mounted onto microscope slides with fluoromount and DAPI (4′,6-diamidino-2-phenylindole). The slides were placed in the fridge for a minimum of 24 hours before imaging on a Delta Vision microscope. The vacuole sizes were calculated using ImageJ software.

### *In vitro* bradyzoite viability assay

The protocol for *in vitro* viability assay was adapted from Zwicker et al. (52). Confluent HFF monolayers (preincubated for 2 hours with 3 µM compound 1) were infected with 5 × 10^3^ tachyzoites of the ME49 (type 2) strain. After 24 hours, the compound 1 was removed to avoid toxicity to the host cells. The cells were allowed to grow and differentiate in a 37°C incubator with ambient CO_2_ for 3 days. The cysts were then incubated with drugs at three times and five times their EC_50_ for an additional 4 days at ambient CO_2_. After 7 days of differentiation, the cysts were collected by scraping the monolayers, syringe lysing the parasites, and treatment with acid/pepsin (170 mM sodium chloride, 60 mM hydrochloric acid, and 0.1 mg/mL pepsin) for 30 minutes in a 37°C water bath. The cysts were then neutralized with 94 mM sodium carbonate (Na_2_CO_3_) and enumerated. Ten thousand bradyzoites were plated per well in a six-well plate and allowed to grow undisturbed in a 37°C incubator with 5% CO_2_ for 14 or 16 days. At this time, cultures were fixed with 100% ethanol and stained with 2.5× crystal violet. The control DMSO plaque image is duplicated from Fig. 1E to be used as the representative image of plaques in Fig. 1D. Plaques were quantified using a light microscope using the 4× objective.

### *Ex vivo* bradyzoite growth and viability assay

Brains of chronically infected mice were collected, homogenized with seven strokes in a glass homogenizer, and then enumerated on a Delta Vision microscope [protocol adapted from reference (45)]. Twenty-five cysts per plate were then placed in a tube for acid/pepsin (170 mM sodium chloride, 60 mM hydrochloric acid, and 0.1 mg/mL pepsin) treatment for 10 minutes in a 37°C water bath. The cysts were then neutralized with 94 mM Na_2_CO_3_. The liberated bradyzoites were then equally distributed into a 12-well plate. For the growth assay, the cells were grown in the presence of compounds or DMSO for 12 and 14 days undisturbed. For the viability assays, the cells were treated with compounds or DMSO for 4 hours before being washed with 1× PBS and then grown for 12 and 14 days in media without compounds. After 12 and 14 days, the cells were fixed with 100% ethanol and stained with 2.5× crystal violet. The control DMSO plaque image is duplicated from Fig. 2B to be used as the representative image of plaques in Fig. 2A. Plaques were quantified on a light microscope using the 4× objective and the size of the plaques was measured using Fiji (ImageJ) software.

### Treatment of mice chronically infected with *T. gondii*

CBA/j female mice (Jackson Laboratory) were injected i.p. with a 200 µL suspension of 500 tachyzoites of the Me49-RFP strain *T. gondii* (type II genotype). About 15% of the mice succumbed to the acute infection before 28 days post-infection. We found that this mortality rate was decreased when infecting with tachyzoites that were recently passaged through mice (by collecting tissue cysts and putting them in culture before reinfecting). BALB/c male mice (Jackson Laboratory) were injected i.p. with a 200 µL suspension of 1,500 tachyzoites of the Pru-GFP strain *T. gondii* (type II genotype) (a gift from Laura Knoll). About 15% of the mice succumbed to the acute infection before 28 days post-infection. Interestingly, if we passed the Pru strain through mice, they became more virulent and about 50% of the mice succumbed to the initial acute infection. At 4 weeks post-infection, the brains of two mice were collected to check for the presence of cysts and to confirm that the mice were in the chronic stage. At this time, the remaining mice were injected i.p. daily for 16 consecutive days with 200 µL solvent (10% Kolliphor HS-15) or compounds dissolved in 10% HS-15. ATQ was administered at 5 mg/kg, and BPH-1218 and BPH-1236 were administered at 1 mg/kg. Mice were euthanized humanely 2 weeks after the final injection. The mouse brains were collected in 1 mL sterile PBS, minced with scissors, and homogenized using a glass homogenizer. Four 20 µL samples (80 µL total or 8% of sample) of each brain homogenate were placed on a glass microscope slide. Cysts were enumerated using a Delta Vision fluorescence microscope. Chronic infection of male Swiss Webster mice followed a similar timeline to the one with the CBA/j mice but infecting with 5,000 tachyzoites of the Me49-RFP strain.

### RT-PCR for quantification of parasite number during *in vivo* chronic infections

Total RNA was extracted from half brains of mice using TRIzol Reagent (Fisher cat # NC0151218) and treated with DNase. The RNA was then reverse-transcribed into cDNA using the cDNA first-strand synthesis protocol using Superscript III RT (Thermo Fisher cat # 18080044) followed by treatment with RNase H. The qPCR was done using the iQ SYBR Green Master Mix (BioRad), plus primers for tubulin (F: GACGACGCCTTCAACACCTTCTTT, R: AGTTGTTCGCAGCATCCTCTTTCC), and the reverse-transcribed cDNA. The qRT-PCR was carried out on a CFX96 PCR Real-Time Detection System (C1000 Touch thermal cycler, BioRad). Relative quantification software (CFX Maestro Software) was used for the analysis, and the relative expression levels were calculated as the fold change using the 2^ΔΔ^CT (53). Expression levels were normalized to parasite number based on the *in vitro* growth of Pru-GFP tachyzoites and the actin (F: TCCACCATGAAGATCAAGGTCGTT, R: ACATCTGCTGGAAGGTGGAG) and tubulin primers (noted above). Experiments were run in triplicate within the plate and repeated for each of the three biological replicates.

### Mouse activity monitoring

Mice were monitored during the infection using the CageDot (30, 31), which detects vibrations. The system is composed of one Raspberry Pi as an on-board computer, one Analog-Digital Converter board, and one vertical Geophone sensor as vibration detector. The Geophone sensor can detect mini vibrations of its attached surface. The raw vibration data are preprocessed to remove noise, and a machine learning model estimates the activity of animals by using extracted features from these data. To achieve minimal influence of noise on subject animals, magnets are used to attach the seismic sensor beneath the experiment cage. Data are collected at a 100 Hz sampling rate (each second, 100 data points were collected). Our system is not only cost-effective but also energy-aware and requires minimal maintenance.

### Cytotoxicity on HFF and C2C12 myoblast cells

The cytotoxicity was tested using an Alamar Blue assay as described by Recher et al. (49). Toxicity was not high enough at the concentrations of drugs tested for detection with Alamar Blue.

### Statistical analysis

Experimental data are expressed as the mean values ± standard error of the mean from at least three independent biological replicates unless indicated otherwise. Statistical analyses were performed using Student’s *t*-test using GraphPad Prism version 9 for experiments with two experimental conditions. For experiments with three or more conditions, a two-way analysis of variance statistical analysis was used (also stated in the figure legends). The results of the *in vivo* studies were also analyzed using a power calculation using R software. A *P*-value of <0.05 was considered statistically significant.

## Data Availability

Data will be made available upon request.

## SUPPLEMENTAL MATERIAL

Supplemental material mbio.01756-24-s0001.pdf Supplemental figures and table.

ASM does not own the copyrights to Supplemental Material that may be linked to, or accessed through, an article. The authors have granted ASM a non-exclusive, world-wide license to publish the Supplemental Material files. Please contact the corresponding author directly for reuse.

## References

[1] Hill D, Dubey JP. 2002. Toxoplasma gondii: transmission, diagnosis and prevention. Clin Microbiol Infect 8:634–640. doi:10.1046/j.1469-0691.2002.00485.x 12390281

[2] Parise ME, Hotez PJ, Slutsker L. 2014. Neglected parasitic infections in the United States: needs and opportunities. Am J Trop Med Hyg 90:783–785. doi:10.4269/ajtmh.13-0727 24808243 PMC4015562

[3] Weiss LM, Kim K. 2000. The development and biology of bradyzoites of Toxoplasma gondii*.* Front Biosci 5:D391–D405. doi:10.2741/weiss 10762601 PMC3109641

[4] Cabral CM, Tuladhar S, Dietrich HK, Nguyen E, MacDonald WR, Trivedi T, Devineni A, Koshy AA. 2016. Neurons are the primary target cell for the brain-tropic intracellular parasite Toxoplasma gondii. PLoS Pathog 12:e1005447. doi:10.1371/journal.ppat.1005447 26895155 PMC4760770

[5] Martinez VO, de Mendonça Lima FW, de Carvalho CF, Menezes-Filho JA. 2018. Toxoplasma gondii infection and behavioral outcomes in humans: a systematic review. Parasitol Res 117:3059–3065. doi:10.1007/s00436-018-6040-2 30109417

[6] Ihara F, Nishimura M, Muroi Y, Mahmoud ME, Yokoyama N, Nagamune K, Nishikawa Y. 2016. Toxoplasma gondii infection in mice impairs long-term fear memory consolidation through dysfunction of the cortex and amygdala. Infect Immun 84:2861–2870. doi:10.1128/IAI.00217-16 27456832 PMC5038065

[7] Hutchinson WM, Bradley M, Cheyne WM, Wells BW, Hay J. 1980. Behavioural abnormalities in Toxoplasma-infected mice. Ann Trop Med Parasitol 74:337–345. doi:10.1080/00034983.1980.11687350 7396566

[8] Vyas A, Kim SK, Giacomini N, Boothroyd JC, Sapolsky RM. 2007. Behavioral changes induced by Toxoplasma infection of rodents are highly specific to aversion of cat odors. Proc Natl Acad Sci U S A 104:6442–6447. doi:10.1073/pnas.0608310104 17404235 PMC1851063

[9] Schoondermark-van de Ven E, Vree T, Melchers W, Camps W, Galama J. 1995. In vitro effects of sulfadiazine and its metabolites alone and in combination with pyrimethamine on Toxoplasma gondii. Antimicrob Agents Chemother 39:763–765. doi:10.1128/AAC.39.3.763 7793889 PMC162621

[10] Doggett J Stone, Nilsen A, Forquer I, Wegmann KW, Jones-Brando L, Yolken RH, Bordón C, Charman SA, Katneni K, Schultz T, Burrows JN, Hinrichs DJ, Meunier B, Carruthers VB, Riscoe MK. 2012. Endochin-like quinolones are highly efficacious against acute and latent experimental toxoplasmosis. Proc Natl Acad Sci U S A 109:15936–15941. doi:10.1073/pnas.1208069109 23019377 PMC3465437

[11] Doggett J.S, Schultz T, Miller AJ, Bruzual I, Pou S, Winter R, Dodean R, Zakharov LN, Nilsen A, Riscoe MK, Carruthers VB. 2020. Orally bioavailable endochin-like quinolone carbonate ester prodrug reduces Toxoplasma gondii brain cysts. Antimicrob Agents Chemother 64:e00535-20. doi:10.1128/AAC.00535-20 32540978 PMC7449172

[12] Martynowicz J, Doggett JS, Sullivan WJ. 2020. Efficacy of guanabenz combination therapy against chronic toxoplasmosis across multiple mouse strains. Antimicrob Agents Chemother 64:e00539-20. doi:10.1128/AAC.00539-20 32540979 PMC7449173

[13] Watts NB. 1998. Treatment of osteoporosis with bisphosphonates. Endocrinol Metab Clin North Am 27:419–439. doi:10.1016/s0889-8529(05)70014-1 9669147

[14] Oldfield E. 2010. Targeting isoprenoid biosynthesis for drug discovery: bench to bedside. Acc Chem Res 43:1216–1226. doi:10.1021/ar100026v 20560544 PMC2943567

[15] Galaka T, Ferrer Casal M, Storey M, Li C, Chao MN, Szajnman SH, Docampo R, Moreno SNJ, Rodriguez JB. 2017. Antiparasitic activity of sulfur- and fluorine-containing bisphosphonates against trypanosomatids and apicomplexan parasites. Molecules 22:82. doi:10.3390/molecules22010082 28054995 PMC6155738

[16] Szajnman SH, Galaka T, Li Z-H, Li C, Howell NM, Chao MN, Striepen B, Muralidharan V, Moreno SNJ, Rodriguez JB. 2017. In vitro and in vivo activities of sulfur-containing linear bisphosphonates against apicomplexan parasites. Antimicrob Agents Chemother 61:e01590-16. doi:10.1128/AAC.01590-16 27895021 PMC5278718

[17] Ling Y, Sahota G, Odeh S, Chan JMW, Araujo FG, Moreno SNJ, Oldfield E. 2005. Bisphosphonate inhibitors of Toxoplasma gondi growth: in vitro, QSAR, and in vivo investigations. J Med Chem 48:3130–3140. doi:10.1021/jm040132t 15857119

[18] Mukkamala D, No JH, Cass LM, Chang TK, Oldfield E. 2008. Bisphosphonate inhibition of a Plasmodium farnesyl diphosphate synthase and a general method for predicting cell-based activity from enzyme data. J Med Chem 51:7827–7833. doi:10.1021/jm8009074 19053772 PMC2765246

[19] Li Z-H, Ramakrishnan S, Striepen B, Moreno SNJ. 2013. Toxoplasma gondii relies on both host and parasite isoprenoids and can be rendered sensitive to atorvastatin. PLoS Pathog 9:e1003665. doi:10.1371/journal.ppat.1003665 24146616 PMC3798403

[20] Sleda MA, Li Z-H, Behera R, Baierna B, Li C, Jumpathong J, Malwal SR, Kawamukai M, Oldfield E, Moreno SNJ. 2022. The heptaprenyl diphosphate synthase (Coq1) Is the target of a lipophilic bisphosphonate that protects mice against Toxoplasma gondii Infection. MBio 13:e0196622. doi:10.1128/mbio.01966-22 36129297 PMC9600589

[21] Xia Y, Liu YL, Xie Y, Zhu W, Guerra F, Shen S, Yeddula N, Fischer W, Low W, Zhou X, Zhang Y, Oldfield E, Verma IM. 2014. A combination therapy for KRAS-driven lung adenocarcinomas using lipophilic bisphosphonates and rapamycin. Sci Transl Med 6:263ra161. doi:10.1126/scitranslmed.3010382 PMC4326221 25411474

[22] Russell RGG. 2011. Bisphosphonates: the first 40 years. Bone 49:2–19. doi:10.1016/j.bone.2011.04.022 21555003

[23] Zhang Y, Cao R, Yin F, Hudock MP, Guo R-T, Krysiak K, Mukherjee S, Gao Y-G, Robinson H, Song Y, No JH, Bergan K, Leon A, Cass L, Goddard A, Chang T-K, Lin F-Y, Beek EV, Papapoulos S, Wang AH-J, Kubo T, Ochi M, Mukkamala D, Oldfield E. 2009. Lipophilic bisphosphonates as dual farnesyl/geranylgeranyl diphosphate synthase inhibitors: An X-ray and NMR investigation. J Am Chem Soc 131:5153–5162. doi:10.1021/ja808285e 19309137 PMC2753403

[24] McFadden DC, Tomavo S, Berry EA, Boothroyd JC. 2000. Characterization of cytochrome B from Toxoplasma gondii and Q(o) domain mutations as a mechanism of atovaquone-resistance. Mol Biochem Parasitol 108:1–12. doi:10.1016/s0166-6851(00)00184-5 10802314

[25] Ferguson DJ, Huskinson-Mark J, Araujo FG, Remington JS. 1994. An ultrastructural study of the effect of treatment with atovaquone in brains of mice chronically infected with the ME49 strain of Toxoplasma gondii*.* Int J Exp Pathol 75:111–116. 8199003 PMC2002108

[26] Radke JR, Donald RG, Eibs A, Jerome ME, Behnke MS, Liberator P, White MW. 2006. Changes in the expression of human cell division autoantigen-1 influence Toxoplasma gondii growth and development. PLoS Pathog 2:e105. doi:10.1371/journal.ppat.0020105 17069459 PMC1626100

[27] Christiansen C, Maus D, Hoppenz E, Murillo-León M, Hoffmann T, Scholz J, Melerowicz F, Steinfeldt T, Seeber F, Blume M. 2022. In vitro maturation of Toxoplasma gondii bradyzoites in human myotubes and their metabolomic characterization. Nat Commun 13:1168. doi:10.1038/s41467-022-28730-w 35246532 PMC8897399

[28] Paredes-Santos TC, Martins-Duarte ES, Vitor RWA, de Souza W, Attias M, Vommaro RC. 2013. Spontaneous cystogenesis in vitro of a Brazilian strain of Toxoplasma gondii. Parasitol Int 62:181–188. doi:10.1016/j.parint.2012.12.003 23269201

[29] Paredes-Santos TC, Tomita T, Yan Fen M, de Souza W, Attias M, Vommaro RC, Weiss LM. 2016. Development of dual fluorescent stage specific reporter strain of Toxoplasma gondii to follow tachyzoite and bradyzoite development in vitro and in vivo. Microbes Infect 18:39–47. doi:10.1016/j.micinf.2015.09.016 26432517 PMC4715970

[30] Pitafi ZF. 2023. Contactless animal activity and behavior monitoring. IEEE. doi:10.1109/PerComWorkshops56833.2023.10150347

[31] Pitafi ZF, Sleda M, Moreno SNJ, Tompkins AM, Brainard BM, Song W. 2024. CageDot: contactless animal activity monitoringsystem to follow infectious disease progress. 2024 IEEE International Conference on Communications (ICC): SAC E-Health Track.

[32] Virus MA, Ehrhorn EG, Lui LM, Davis PH. 2021. Neurological and neurobehavioral disorders associated with Toxoplasma gondii infection in humans. J Parasitol Res 2021:6634807. doi:10.1155/2021/6634807 34712493 PMC8548174

[33] Place BC, Troublefield CA, Murphy RD, Sinai AP, Patwardhan AR. 2023. Machine learning based classification of mitochondrial morphologies from fluorescence microscopy images of Toxoplasma gondii cysts. PLoS One 18:e0280746. doi:10.1371/journal.pone.0280746 36730225 PMC9894464

[34] Li Z-H, Cintrón R, Koon NA, Moreno SNJ. 2012. The N-terminus and the chain-length determination domain play a role in the length of the isoprenoid product of the bifunctional Toxoplasma gondii farnesyl diphosphate synthase. Biochemistry 51:7533–7540. doi:10.1021/bi3005335 22931372 PMC4618988

[35] Ling Y, Li ZH, Miranda K, Oldfield E, Moreno SNJ. 2007. The farnesyl-diphosphate/geranylgeranyl-diphosphate synthase of Toxoplasma gondii is a bifunctional enzyme and a molecular target of bisphosphonates. J Biol Chem 282:30804–30816. doi:10.1074/jbc.M703178200 17724033

[36] Li ZH, Li C, Szajnman SH, Rodriguez JB, Moreno SNJ. 2017. Synergistic activity between statins and bisphosphonates against acute experimental toxoplasmosis. Antimicrob Agents Chemother 61. doi:10.1128/AAC.02628-16 PMC5527628 28559264

[37] Mukherjee S, Huang C, Guerra F, Wang K, Oldfield E. 2009. Thermodynamics of bisphosphonates binding to human bone: a two-site model. J Am Chem Soc 131:8374–8375. doi:10.1021/ja902895p 19489581 PMC2753405

[38] Araujo FG, Shepard RM, Remington JS. 1991. In vivo activity of the macrolide antibiotics azithromycin, roxithromycin and spiramycin against Toxoplasma gondii. Eur J Clin Microbiol Infect Dis 10:519–524. doi:10.1007/BF01963942 1655433

[39] Araujo FG, Huskinson J, Remington JS. 1991. Remarkable in vitro and in vivo activities of the hydroxynaphthoquinone 566C80 against tachyzoites and tissue cysts of Toxoplasma gondii. Antimicrob Agents Chemother 35:293–299. doi:10.1128/AAC.35.2.293 2024964 PMC244994

[40] Dunay IR, Heimesaat MM, Bushrab FN, Müller RH, Stocker H, Arasteh K, Kurowski M, Fitzner R, Borner K, Liesenfeld O. 2004. Atovaquone maintenance therapy prevents reactivation of toxoplasmic encephalitis in a murine model of reactivated toxoplasmosis. Antimicrob Agents Chemother 48:4848–4854. doi:10.1128/AAC.48.12.4848-4854.2004 15561866 PMC529229

[41] Romand S, Pudney M, Derouin F. 1993. In vitro and in vivo activities of the hydroxynaphthoquinone atovaquone alone or combined with pyrimethamine, sulfadiazine, clarithromycin, or minocycline against Toxoplasma gondii. Antimicrob Agents Chemother 37:2371–2378. doi:10.1128/AAC.37.11.2371 8285620 PMC192394

[42] Katlama C, Mouthon B, Gourdon D, Lapierre D, Rousseau F. 1996. Atovaquone as long-term suppressive therapy for toxoplasmic encephalitis in patients with AIDS and multiple drug intolerance. Atovaquone expanded access group. AIDS 10:1107–1112. 8874627

[43] Pearson PA, Piracha AR, Sen HA, Jaffe GJ. 1999. Atovaquone for the treatment of Toxoplasma retinochoroiditis in immunocompetent patients. Ophthalmology 106:148–153. doi:10.1016/S0161-6420(99)90021-0 9917796

[44] Sullivan WJ, Jeffers V. 2012. Mechanisms of Toxoplasma gondii persistence and latency. FEMS Microbiol Rev 36:717–733. doi:10.1111/j.1574-6976.2011.00305.x 22091606 PMC3319474

[45] Radke JB, Melillo B, Mittal P, Sharma M, Sharma A, Fu Y, Uddin T, Gonse A, Comer E, Schreiber SL, Gupta AK, Chatterjee AK, Sibley LD. 2022. Bicyclic azetidines target acute and chronic stages of Toxoplasma gondii by inhibiting parasite phenylalanyl t-RNA synthetase. Nat Commun 13:459. doi:10.1038/s41467-022-28108-y 35075105 PMC8786932

[46] Johnson HJ, Koshy AA. 2020. Latent toxoplasmosis effects on rodents and humans: how much is real and how much is media hype? MBio 11:e02164-19. doi:10.1128/mBio.02164-19 32184245 PMC7078474

[47] Vella SA, Calixto A, Asady B, Li ZH, Moreno SNJ. 2020. Genetic indicators for calcium signaling studies in Toxoplasma gondii. Methods Mol Biol 2071:187–207. doi:10.1007/978-1-4939-9857-9_11 31758454 PMC7294879

[48] van Dooren GG, Tomova C, Agrawal S, Humbel BM, Striepen B. 2008. Toxoplasma gondii Tic20 is essential for apicoplast protein import. Proc Natl Acad Sci U S A 105:13574–13579. doi:10.1073/pnas.0803862105 18757752 PMC2533231

[49] Recher M, Barboza AP, Li Z-H, Galizzi M, Ferrer-Casal M, Szajnman SH, Docampo R, Moreno SNJ, Rodriguez JB. 2013. Design, synthesis and biological evaluation of sulfur-containing 1,1-bisphosphonic acids as antiparasitic agents. Eur J Med Chem 60:431–440. doi:10.1016/j.ejmech.2012.12.015 23318904 PMC3582829

[50] Radke JR, Behnke MS, Mackey AJ, Radke JB, Roos DS, White MW. 2005. The transcriptome of Toxoplasma gondii. BMC Biol 3:26. doi:10.1186/1741-7007-3-26 16324218 PMC1325263

[51] Tobin C, Pollard A, Knoll L. 2010. Toxoplasma gondii cyst wall formation in activated bone marrow-derived macrophages and bradyzoite conditions. J Vis Exp 42:2091–2094. doi:10.3791/2091 PMC3156017 20736916

[52] Zwicker JD, Smith D, Guerra AJ, Hitchens JR, Haug N, Vander Roest S, Lee P, Wen B, Sun D, Wang L, Keep RF, Xiang J, Carruthers VB, Larsen SD. 2020. Discovery and optimization of triazine nitrile inhibitors of Toxoplasma gondii cathepsin L for the potential treatment of chronic toxoplasmosis in the CNS. ACS Chem Neurosci 11:2450–2463. doi:10.1021/acschemneuro.9b00674 32027110 PMC7431380

[53] Livak KJ, Flood SJ, Marmaro J, Giusti W, Deetz K. 1995. Oligonucleotides with fluorescent dyes at opposite ends provide a quenched probe system useful for detecting PCR product and nucleic acid hybridization. PCR Methods Appl 4:357–362. doi:10.1101/gr.4.6.357 7580930

