# Supplementary material for: Lipophilic bisphosphonates reduced cyst burden and ameliorated hyperactivity of mice chronically infected with Toxoplasma gondii
Source: mBio. 2024 Oct 10;15(11):e01756-24. doi: 10.1128/mbio.01756-24 (PMC11558998; doi:10.1128/mbio.01756-24)
Supplement: Supplemental material — Supplemental figures and table. [file mbio.01756-24-s0001.pdf]

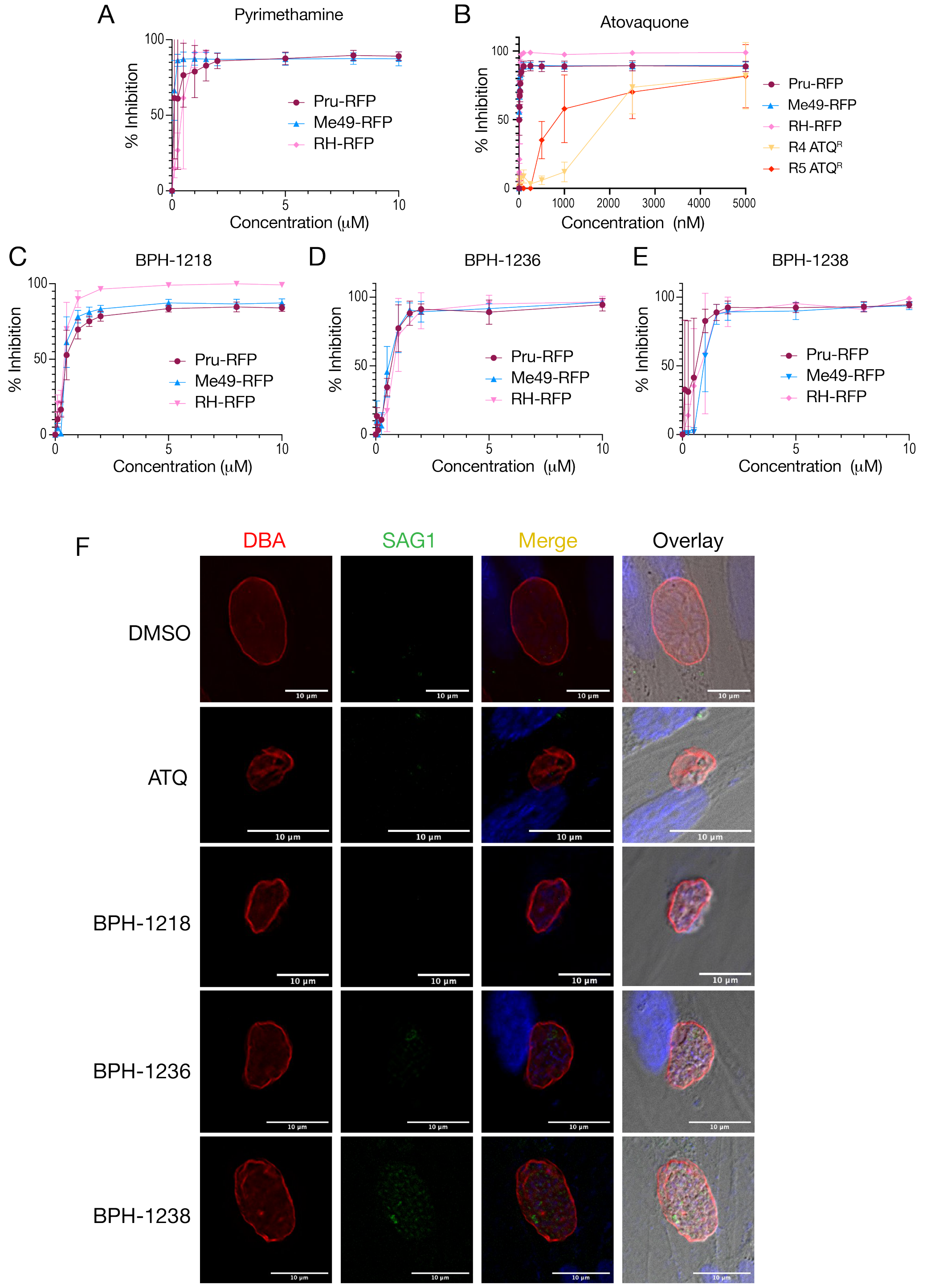

Figure S1. Bisphosphonates can inhibit growth of tachyzoites from various strains of *Toxoplasma gondii*. EC_{50} curves of Pyrimethamine (A), Atovaquone (B), BPH-1218 (C), BPH-1236 (D), and BPH-1238 (E) for the RH, Pru, Me49, and ATQ^R strains. All data shown are the averages of 3 biological replicates. (F) Representative images from *in vitro* bradyzoite IFA at 3X EC_{50}.

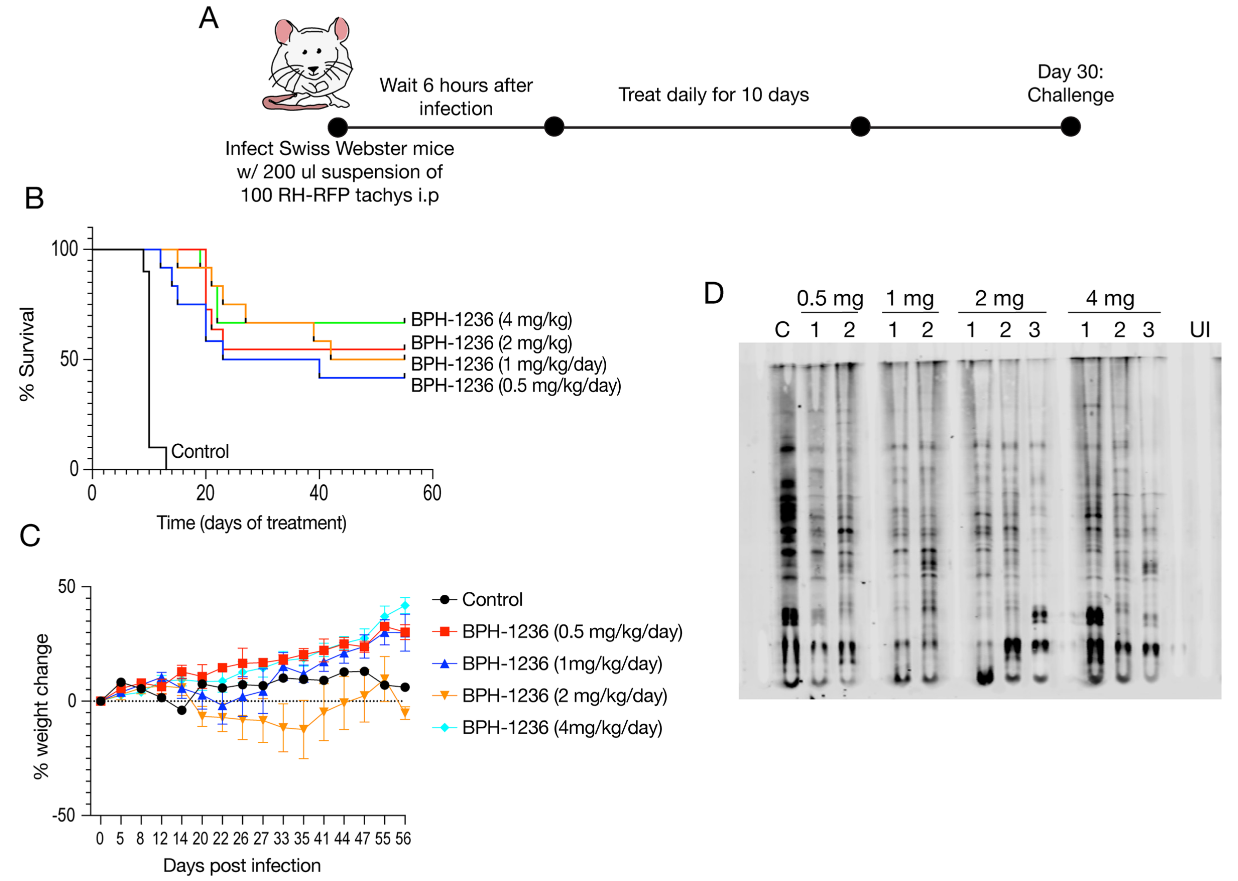

Figure S2. Bisphosphonates can inhibit the growth of tachyzoites in various strains of *Toxoplasma gondii*. A. Model of acute infection. Swiss webster mice are infected i.p. with 100 RH-RFP tachyzoites and 6 hours later daily i.p. treatment is started at the indicated concentrations. All concentrations are in mg/kg/day. B. Treatment with BPH-1236 had 4 mice for each of 3 biological replicates, therefore each line represents data from 12 mice. Except for the 2 mg/kg group which has 11 mice because 1 mouse had to be sacrificed for reasons unrelated to the *Toxoplasma* infection. C. Average percent change in weight from day 0 of the experiment for the acute infections with BPH-1236. Weights are the averages from 3 independent biological experiments. D. Representative western blots for mouse serum of blood collected at 28 dpi to verify mouse infection before challenging. Blots were tested against total lysate of protein from the RH-RFP tachyzoite strain that was used for infection. Serums were tested at 1:500 dilution.

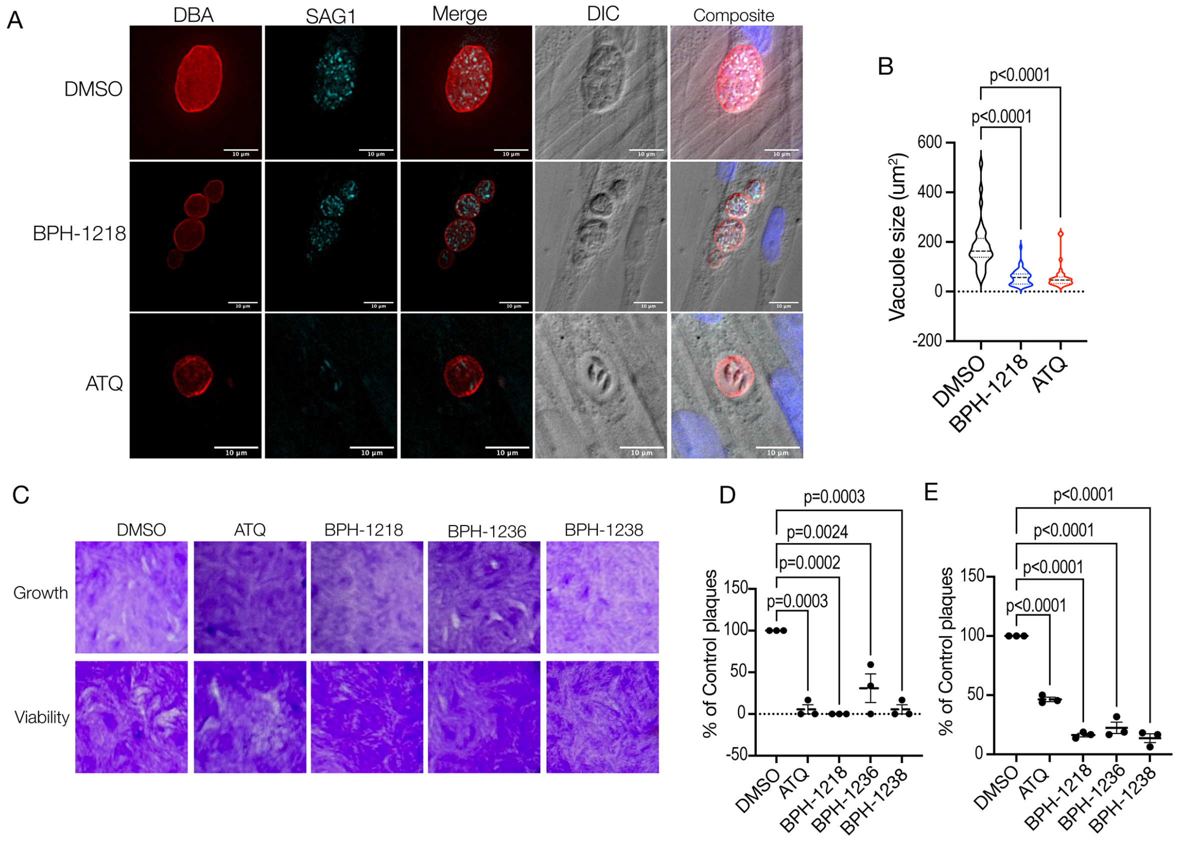

Figure S3. Effects of bisphosphonates on the *T. gondii* Pru strain.

A. Representative images of *in vitro* derived cysts with DBA (cyst wall) and SAG1 (tachyzoite marker) staining. B. Quantification of the vacuole size of the cysts in A. C. Representative images from the *ex vivo* growth and viability of Pru-GFP. D. Quantification of the number of plaques from the *ex vivo* growth of Pru-GFP. E. Quantification of the number of plaques from the *ex vivo* viability of Pru-GFP. Data from B, D, and E are averages from 3 independent biological replicates. Two-way anova statistical analysis was performed.

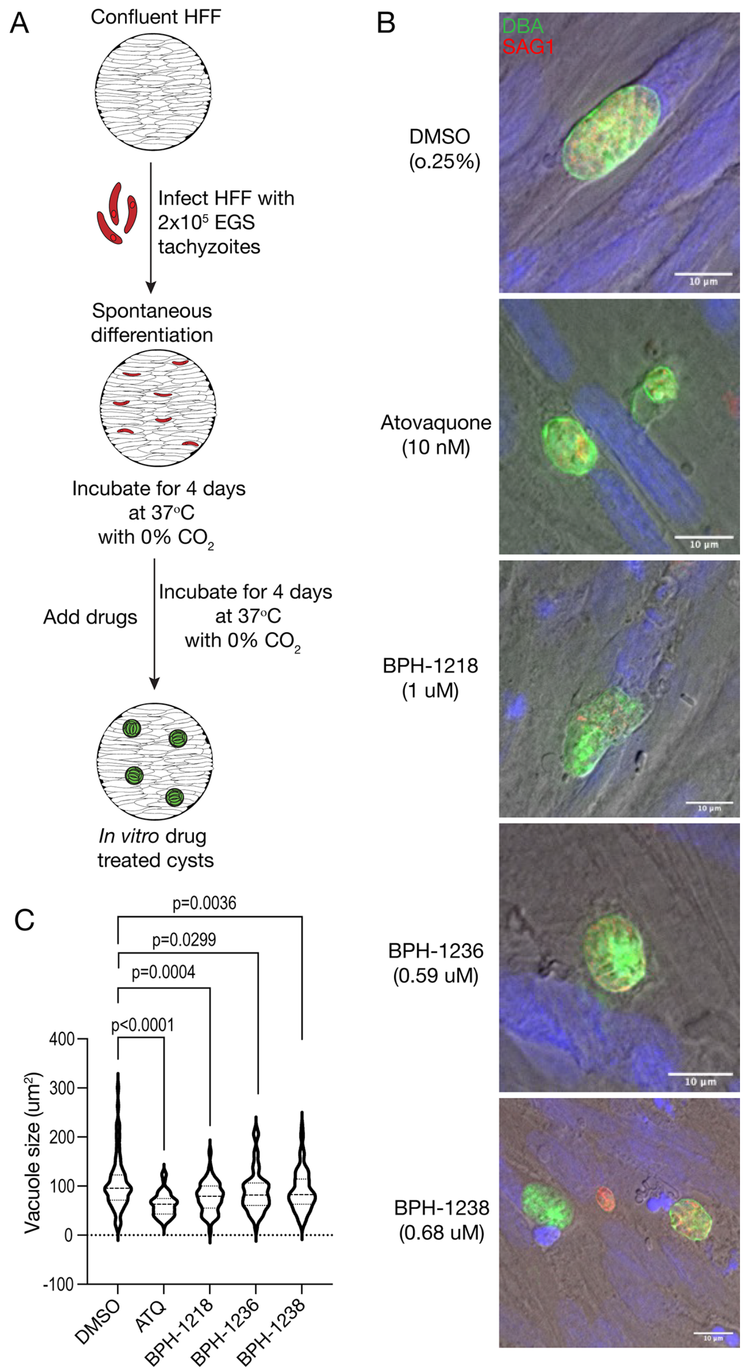

Figure S4. Effects of bisphosphonates on a type I/III strain of bradyzoites.

A. Schematic representation of spontaneous differentiation in the type III strain EGS. B. Representative images of EGS drug treated cysts. C. Quantification of the vacuole size from 3 independent biological replicates. Two-way anova statistical analysis was performed.

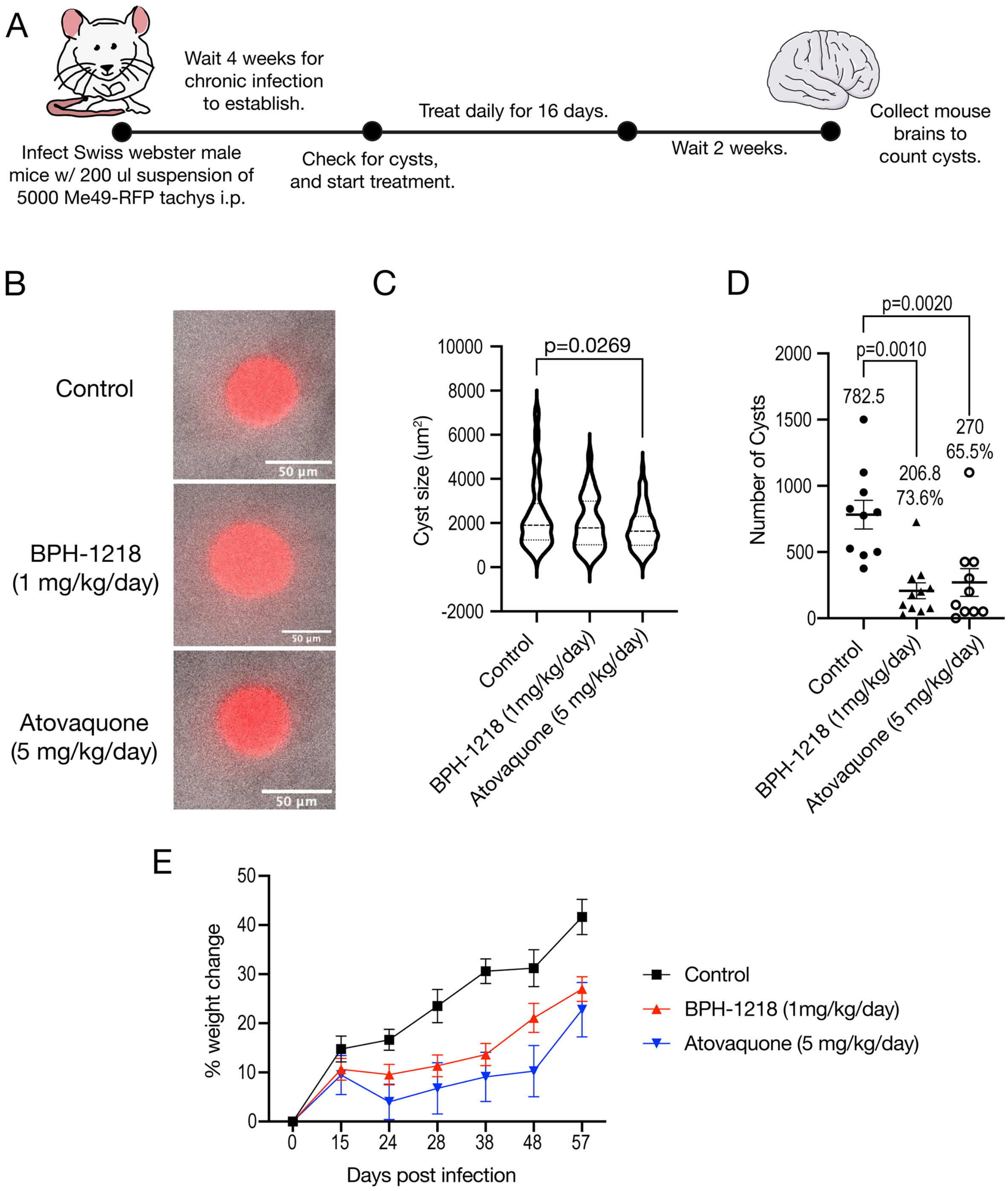

Figure S5. Bisphosphonates protect Swiss webster mice chronically infected with Me49-RFP. A. Schematic representation of the chronic infection. Swiss webster mice are infected i.p. with 5000 Me49-RFP tachyzoites, after 4 weeks the mice were treated daily for 16 days with the indicated concentrations of drugs. Two weeks after treatment the brains from all mice were collected and cysts enumerated. B. Representative images of cysts from control and drug treated mice. C. Quantification of the cyst size from 3 biological replicates. D. Quantification of the number of cysts from 3 biological replicates. E. The percent of weight change through the experiments, an average from 3 biological replicates. Two-way anova statistical analysis was performed for C and D.

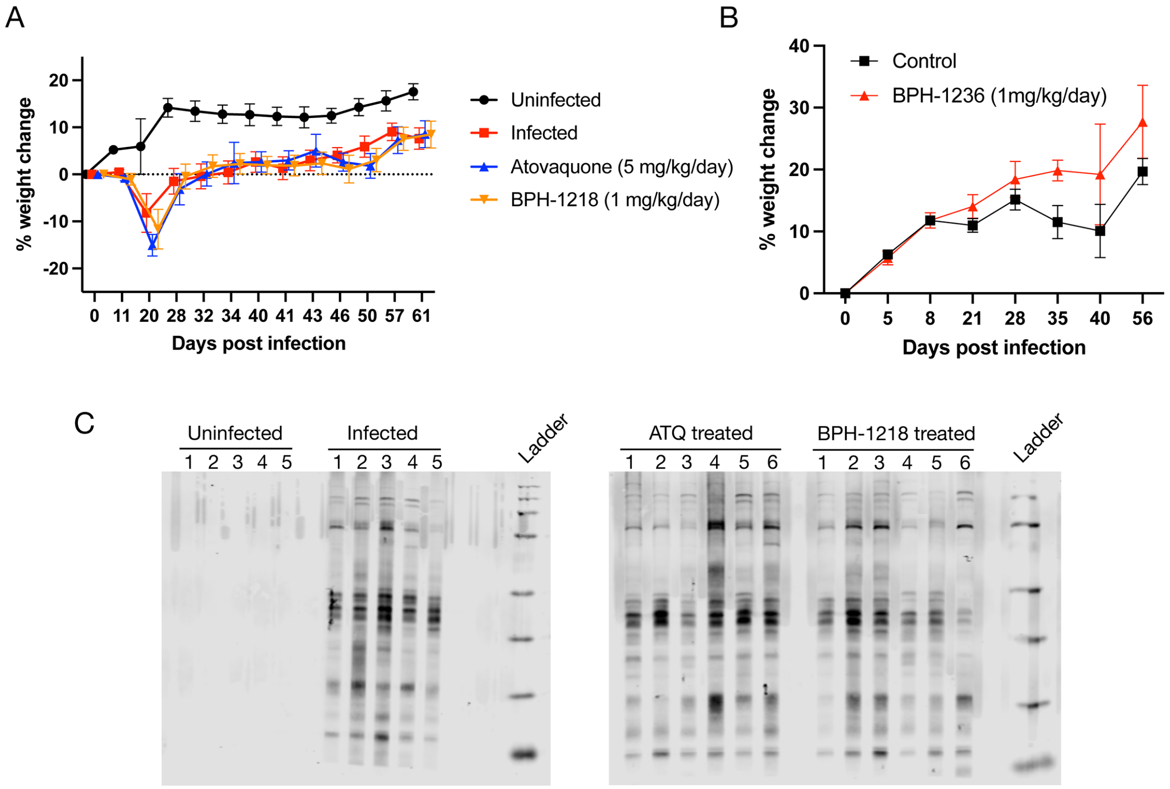

Figure S6. Mouse weight data and western blots of mouse serum.

A. Average weights for Balb/c mice infected with Pru-GFP for the chronic infection activity experiment. Data is the average from 3 independent biological experiments. B. Average weights for CBA/j mice infected with Me49-RFP for chronic infections. The data is the average for all mice from 3 independent biological replicates. C. Representative western blots of mouse serum tested against total lysate of Pru-GFP protein to ensure infection at the end of experiment (day 56).

Table S1. Cytotoxicity in Human Foreskin Fibroblasts (HFF) and C2C12 myoblasts.

Drug (X EC_{50})	% Inhibition on HFF	% Inhibition on C2C12
Atovaquone 4X	4.69 ± 2.56	2.61 ± 4.48
Atovaquone 10X	7.23 ± 2.29	6.84 ± 5.34
Pyrimethamine 4X	0.38 ± 2.61	2.39 ± 3.50
Pyrimethamine 10X	6.18 ± 0.98	6.57 ± 1.31
BPH-1218 4X	7.40 ± 1.75	4.18 ± 2.94
BPH-1218 10X	14.32 ± 1.11	10.10 ± 4.94
BPH-1236 4X	6.96 ± 3.16	8.82 ± 5.68
BPH-1236 10X	11.12 ± 1.67	12.06 ± 5.81
BPH-1238 4X	10.11 ± 3.60	4.23 ± 4.18
BPH-1238 10X	17.46 ± 4.05	10.55 ± 3.76
BPH-252 4X	1.87 ± 3.21	6.74 ± 2.67
BPH-252 10X	8.32 ± 1.14	6.74 ± 2.67
